# Preparation of Multifunctional Nano‐Protectants for High‐Efficiency Green Control of Anthracnose

**DOI:** 10.1002/advs.202410585

**Published:** 2024-11-18

**Authors:** Jiaming Yin, Jiajia Zhao, Zeng Wang, Zhen Fang, Huiming Guo, Hongmei Cheng, Jie Li, Jie Shen, Meizhen Yin, Xiaofeng Su, Shuo Yan

**Affiliations:** ^1^ Frontiers Science Center for Molecular Design Breeding Department of Plant Biosecurity College of Plant Protection China Agricultural University Beijing 100193 China; ^2^ Sanya Institute of China Agricultural University Sanya 572025 China; ^3^ National Key Laboratory of Agricultural Microbiology Biotechnology Research Institute Chinese Academy of Agricultural Sciences Beijing 100081 China; ^4^ National Nanfan Research Institute Chinese Academy of Agricultural Sciences Sanya 572000 China; ^5^ State Key Laboratory of Chemical Resource Engineering Beijing Lab of Biomedical Materials Beijing University of Chemical Technology Beijing 100029 China

**Keywords:** drug delivery, fungicidal activity, nanocarrier, nano‐protectant, plant immune

## Abstract

Nanomaterials cannot only act as active ingredients (AIs), but also adjuvants to encapsulate or attach AIs to improve their fungicidal activity. Herein, a hydrophilic and lipophilic diblock polymer (HLDP) is designed and synthesized to prepare a series of HLDP nano‐protectants to explore the best HLDP nano‐protectant for anthracnose management. These results demonstrate that the HLDP‐CS nano‐protectant displays the best control effects on mango anthracnose via the direct pathogen inhibition and amplified plant immune responses. The HLDP can be spontaneously conjugated with CS into nanoscale spherical particles through hydrophobic interaction. The complexation of CS with HLDP remarkably improves the deposition and adhesion of CS droplets on mango leaves. The HLDP can interact with mycelium via electrostatic interaction to damage the cell wall/membrane, which can act as an AI to directly suppress the spore germination and mycelial growth. Meanwhile, HLDP can be applied as an adjuvant for CS to amplify the plant immune responses via accelerating the biosynthesis of secondary metabolites and plant hormones. This work reports the multiple missions for nanomaterials in pathogen control, which proposes a novel strategy for designing nano‐protectant with dual‐synergistic mechanism.

## Graphical abstract

HLDP self‐assembled with CS to form HLDP‐CS nano‐protectant via hydrophobic interaction. The efficiency green control of mango anthracnose is achieved via the direct pathogen inhibition and amplified plant defence responses.

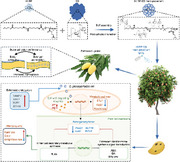

## 1 Introduction

*Colletotrichum* is a cosmopolitan fungal genus comprised of more than 189 species, which has led to significant economic losses in agricultural industry.^[^ ^1^, ^2^, ^3^, ^4^ ^]^ For instance, anthracnose caused by *Colletotrichum gloeosporioides (C. gloeosporioides)* is one of the most important diseases in *Mangifera indica*, which has been estimated to cause economic losses of ≈300 million USD annually in China.^[^ ^5^ ^]^ *C. gloeosporioides* has a latent infection characteristic, where the invasive nails produced by appressorium penetrate the cuticle directly into young fruits, and maintain the latent state in the form of mycelium until the fruits ripen.^[^ ^5^ ^]^ Its effective control is extremely difficult because of the long‐term latency of mycelium, and there are nearly no effective fungicides or resistant germplasms available for controlling *C. gloeosporioides*.^[^ ^6^ ^]^ Traditional pesticides have played an irreplaceable role in prevention and control of plant diseases and insect pests, but their poor dispersibility and large particle in aqueous solution limit their bioactivity and result in low utilization rate in actual production.^[^ ^7^, ^8^, ^9^, ^10^, ^11^ ^]^ Therefore, there is an urgent need to introduce innovative technologies to improve pesticide bioactivity and achieve high utilization rate.

In recent years, nanotechnology has shown great potential in the design and development of novel agrochemicals, which may overcome the limitations of traditional pesticides.^[^ ^12^, ^13^, ^14^, ^15^ ^]^ The primary research hotspots of nanomaterials in disease control include: 1) Nanomaterials can act as active ingredients (AIs) to directly interact with pathogens, leading to the physical adsorption or chemical interactions that render pathogen inactive.^[^ ^16^, ^17^, ^18^, ^19^ ^]^ For instance, the Ag nanoparticles with a size range of 5–50 nm exhibit strong antimicrobial activity against various gram‐positive and gram‐negative bacteria.^[^ ^20^ ^]^ The TiO_2_ nanoparticles display antibacterial activity against *Escherichia coli*, *Staphylococcus aureus* and *Candida albicans*.^[^ ^21^ ^]^ 2) Nanomaterials can be applied as adjuvants to encapsulate or attach AIs to their peripheral groups.^[^ ^22^, ^23^, ^24^, ^25^ ^]^ Its advantages include enhanced stability, improved delivery, sustainable release and elevated efficacy.^[^ ^26^, ^27^ ^]^ Thus, nanotechnology is expected to improve the delivery and bioactivity of protectants, which may meet the actual demands for mango anthracnose management.

Recently, our group has constructed a hydrophilic and lipophilic diblock polymer (HLDP) that shows direct fungicidal activity and high delivery efficiency.^[^ ^28^ ^]^ The HLDP possesses both hydrophilic and hydrophobic groups, which enable it to bind with exogenous substances through hydrophobic interaction, electrostatic interaction, etc. The HLDP can assemble with plant elicitor salicylic acid (SA), and the complexation with HLDP can increase the delivery efficiency and plant uptake of SA, showing great potential in the design of novel nano‐protectants for plant protection. As benzimidazole pesticides, carbendazim (CBZ) and prochloraz (PRO) are registered as fungicides to control mango anthracnose. For instance, CBZ and flutriafol display the best fungicidal activity against *Fusarium incarnatum*, with median effect concentration (EC_50_) values of 0.211 and 0.214 ppm, respectively.^[^ ^29^ ^]^ CBZ can selectively bind to β‐tubulin monomer to disrupt the microtubule polymerization process and damage the cell mitosis, which subsequently leads to the failure of cell division.^[^ ^29^, ^30^, ^31^ ^]^ PRO can interfere with the integrity of fungal cell membrane and inhibit the synthesis of nucleic acids, thus leading to the cell death.^[^ ^32^, ^33^ ^]^ Chitosan (2‐acetamido‐2‐deoxy‐αD‐glucose‐(N‐acetylglucosamine), CS) and SA are representative plant elicitors derived from natural compounds, which can boost the plant immune system to defence against plant pathogens.^[^ ^34^, ^35^, ^36^, ^37^ ^]^ However, the control efficacies of above protectants are very limited toward mango anthracnose, and the complexation with HLDP is expected to overcome their disadvantages, which is conducive to developing high‐efficiency protectants.

The current study aimed to apply HLDP to construct a series of HLDP nano‐protectants based on two plant elicitors (CS and SA) and two fungicides (CBZ and PRO), and compared their bioactivities to screen the best nano‐protectant for controlling mango anthracnose. Herein, the loading efficiencies and the self‐assembly mechanism of HLDP with protectant was elucidated. The uptake of HLDP‐loaded protectants was tested using mango fruits. Subsequently, the direct fungicidal activities of HLDP nano‐protectants against *C. gloeosporioides* were determined and compared, and the potential inhibitory mechanism of HLDP‐CS nano‐protectant with the highest fungicidal activity was investigated. Meanwhile, the control effects of HLDP‐CS nano‐protectant on mango anthracnose was evaluated, and several parameters for fruit quality were also examined. Finally, the differentially expressed genes (DEGs) and differentially expressed metabolites (DEMs) in mango fruits treated with HLDP‐CS nano‐protectant and CS alone were co‐analyzed to illustrate the potential mechanism underlying HLDP‐mediated amplified plant immune responses. Overall, a self‐assembled HLDP‐CS nano‐protectant was successfully developed with a dual synergistic mechanism, which could be applied to control mango anthracnose via the direct pathogen inhibition and amplified plant immune responses.

## 2 Results and Discussion

### 2.1 Self‐Assembly Mechanism of HLDP Nano‐Protectants

**Figure 1:**
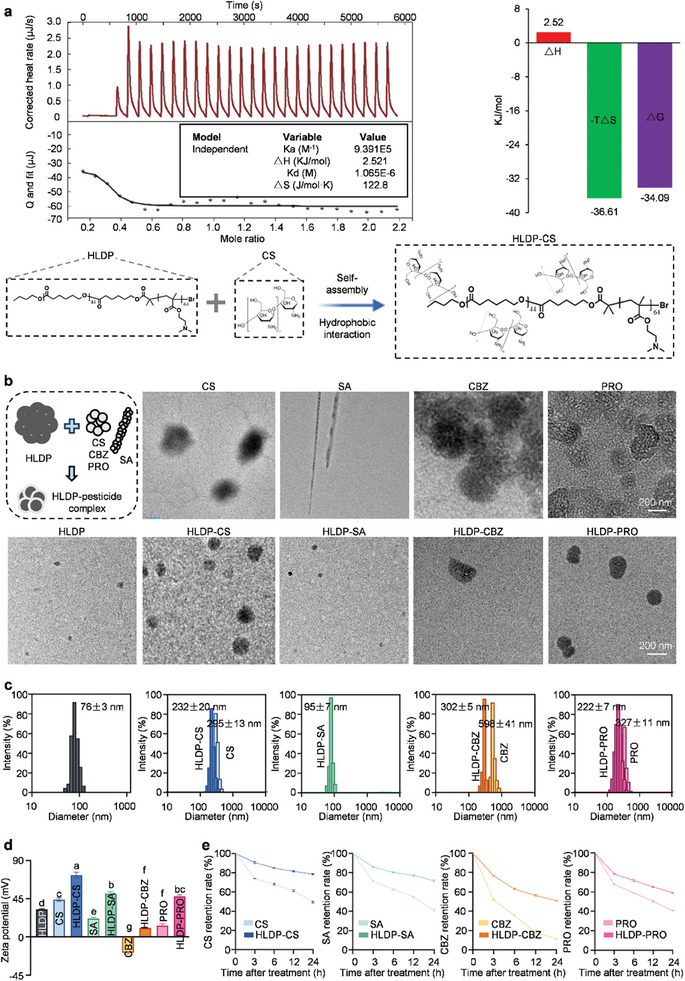
Characterization and properties of HLDP nano‐protectants. a) Isothermal titration calorimetry of CS into HLDP. The 1000 µL of HLDP solution (0.138 mm) was titrated with 250 µL of CS solution (1 mm). During each injection, the heating temperature of interaction was calculated by integrating each titration peak via Origin7 software, and ΔG value was calculated using the formula of ΔG = ΔH – TΔS. HLDP self‐assembled with CS to form HLDP‐CS nano‐protectant via hydrophobic interaction. b) Morphologies of HLDP nano‐protectants (HLDP concentration: 5 mg mL^−1^). c) Particle size distributions of HLDP nano‐protectants (HLDP concentration: 5 mg mL^−1^). Each treatment contained three independent samples. d) Zeta potentials of HLDP nano‐protectants (HLDP concentration: 5 mg mL^−1^). Each treatment contained three independent samples. Different letters indicate significant differences according to the Brown‐Forsythe test (*P* < 0.05). e) Retention rates of HLDP nano‐protectants (HLDP concentration: 5 mg mL^−1^) treated with light. Each treatment included three independent samples.

The standard calibration curves of plant elicitors and fungicides were constructed using the high performance liquid chromatography (HPLC), and the loading efficiencies of HLDP toward CS (51.21 ± 0.7%) and SA (48.49 ± 1.2%) were higher than those toward CBZ (28.25 ± 1.5%) and PRO (29.64 ± 3.0%) (Figures S1 and S2, Supporting Information). The R^2^ values of the CS, SA, CBZ, and PRO standard curves were 0.992, 0.998, 0.997, and 0.999, respectively. By utilizing protectant standards instead of commercial reagents, more precise quantitative determinations could be achieved, facilitating the easier comparisons. The dominant interaction force for the self‐assembly of HLDP nano‐protectants was further analyzed using the isothermal titration calorimetry (ITC) according to the previous interpretation.^[^ ^38^, ^39^ ^]^ As shown in **Figure** **1**a, the high affinity constant Ka and low dissociation constant Kd indicated the effective and strong interaction between HLDP and CS, and the negative ΔG value suggested that this interaction was spontaneous. The positive values of ΔH (2.521 kJ mol) and ΔS (122.8 J mol^−1^ K^−1^) demonstrated that the self‐assembly of HLDP‐CS complex was mainly driven by hydrophobic interaction. Similarly, our results also demonstrated that the HLDP assembled with SA via electrostatic interaction (Figure S3, Supporting Information), CBZ via hydrogen bonding and Van der Waals forces (Figure S4, Supporting Information), and PRO via hydrophobic interaction (Figure S5, Supporting Information). HLDP could assemble with different types of protectants through various interaction forces, which was dependent on many factors, such as hydrophilicity, surface charge, solvent, etc.

The nanomaterial HLDP is consisted of hydrophobic and hydrophilic groups. The hydrophobic carbon chains can be applied to assemble with hydrophobic AIs, and the hydrophilic carboxylic and hydroxyl groups make HLDP more stable in aqueous solution. Thus, the HLDP is expected to assemble with various types of groups and serve as a universal adjuvant. Our group has designed and synthesized a star polyamine (SPc) that functions similarly to HLDP, which has been used as an efficient gene/pesticide nanocarrier.^[^ ^40^ ^]^ The interaction forces between SPc and exogenous substances are diverse, including the hydrogen bonding and Van der Waals forces with thiophanate methyl and chitosan, electrostatic interaction with fluopyram, and hydrophobic interaction with matrine.^[^ ^41^, ^42^, ^43^, ^44^ ^]^

### 2.2 Reduced Particle Size and Enhanced Stability of Protectants after the Complexation with HLDP

Based on the representative transmission electron microscope (TEM) images, the complexation with HLDP formed the stable spherical HLDP nano‐protectants with smaller particle size compared to protectant alone (Figure 1b). All HLDP nano‐protectants were primarily consisted of stable spherical particles. Notably, the complexation with HLDP changed the morphology of SA from needle‐like particles to spherical particles. The particle sizes of CS, CBZ, and PRO were 295 ± 13, 598 ± 41, and 327 ± 11 nm, respectively, and those reduced to 232 ± 20, 302 ± 5, and 222 ± 7 nm with the aid of HLDP (Figure 1c). The HLDP could be applied as an adjuvant to self‐assemble with protectants, and break the aggregated structures of protectants to reduce their particle sizes to nanoscale. Similarly, a previous publication reports that the HLDP can assemble with SA to form a stable complex with the particle size of 67 nm.^[^ ^28^ ^]^ The pesticide nanometerization by polymer, and poly(ethylene glycol)‐poly(D,L‐lactide) can assemble with lambda‐cyhalothrin to decrease its particle size down to nanoscale (258 nm).^[^ ^45^ ^]^ Reduced particle size can not only amplify the contact area of pesticides to harmful organisms, but also improve the distribution and adhesion performance of pesticides on plant surface, thereby increasing the bioactivity.^[^ ^46^, ^47^ ^]^

The HLDP was a cationic polymer with the zeta potential of 32.63 ± 0.7 mV, and the zeta potentials of CS, SA, CBZ, and PRO were 43.97 ± 2.0, 21.80 ± 3.4, −18.73 ± 1.2, and 13.20 ± 2.0 mV, respectively (Figure 1d). As expected, all HLDP nano‐protectants were positively‐charged, and their zeta potentials were higher than those of corresponding protectants, which was attributed to the positive charge of HLDP. Notably, the zeta potential of HLDP‐CS complex was the highest (72.83 ± 3.4 mV) among various HLDP nano‐protectants (*F*_8,18_ = 639.8, *P *< 0.0001). Previous results have shown that nano‐pesticides with high positive charges display strong affinity to biological membrane, thus exhibiting greater inhibitory effects on bacteria.^[^ ^48^, ^49^, ^50^ ^]^ The CS, SA, CBZ, and PRO can be easily degraded under light conditions, leading to structural changes or decomposition that reduce their effectiveness in field applications.^[^ ^51^, ^52^, ^53^, ^54^ ^]^ As shown in Figure 1e, interestingly, the complexation with HLDP remarkably increased their stabilities, and the retention rates of CS, SA, CBA, and PRO increased from 49.53%, 40.97%, 11.27%, and 40.70% to 78.60%, 71.60%, 50.77%, and 59.1% at 24 h after the irradiation, respectively. The HLDP might provide a protective layer/certain physical barrier for protectants to alleviate their degradation.^[^ ^21^, ^55^ ^]^

Particle dissolution plays a significant role in determining the properties and potential applications of nanomaterials.^[^ ^56^, ^57^, ^58^ ^]^ The dissolution of HLDP nano‐protectants was also investigated, and the results revealed that the particle sizes of HLDP‐nano‐protectants were stable under the oscillation condition (Figure S6a, Supporting Information). In addition, the zeta potentials and particle sizes of all HLDP nano‐protectants were relatively stable after the storage at room temperature (Figure S6b, Supporting Information).

### 2.3 Stronger Adhesion Performance and Plant Uptake of Protectants after the Complexation with HLDP

**Figure 2:**
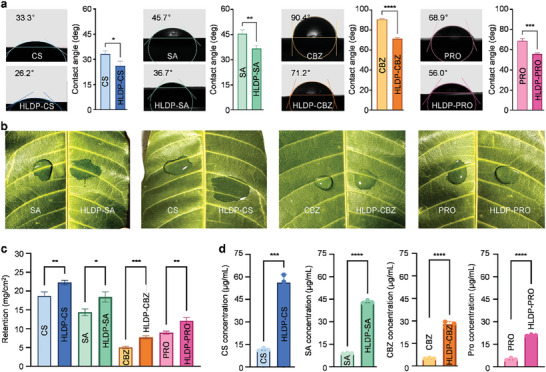
a) Contact angle, b) spreading performance, c) retention, and d) plant uptake of HLDP nano‐protectants. a) Contact angle of HLDP nano‐protectants (protectant concentration: 5 mg mL^−1^) on mango leaves. The 5 µL of each formulation was dripped onto the leaf, and the image was collected. The test was repeated three times for each solution. The “*”, “**”, “***” and “****” indicate significant differences according to the ratio paired t test (*P* < 0.05, *P* < 0.01, *P* < 0.001, and *P* < 0.0001). b) Spreading performance of HLDP nano‐protectants (protectant concentration: 5 mg mL^−1^) on mango leaves. c) Retention of HLDP nano‐protectants (protectant concentration: 5 mg mL^−1^) on mango leaves. Each treatment included eight independent samples. d) Plant uptake of HLDP nano‐protectants (protectant concentration: 5 mg mL^−1^). Each treatment contained three independent samples.

The efficient deposition and strong adhesion of pesticide droplets can reflect the pesticide retention on plant leaves, which is crucial for high‐efficiency utilization. As shown in **Figure** **2**a, the contact angles of CS, SA, CBZ, and PRO were 33.3 ± 1.7°, 45.7 ± 2.0°, 90.4 ± 1.0°, and 68.9 ± 2.1°on mango leaves, respectively, and those reduced to 26.2 ± 2.8°, 36.6 ± 1.9°, 71.1 ± 1.2°, and 56.0 ± 0.9° with the aid of HLDP, indicating that the complexation with HLDP could significantly decrease the contact angles of protectants on plant leaves (CS: *t* = 3.718, df = 4, *P* = 0.025; SA: *t* = 5.685, df = 4, *P* = 0.047; CBZ: *t* = 21.29, df = 4, *P *< 0.0001; PRO: *t* = 9.779, df = 4, *P *= 0.0006). The contact angle of pesticide droplet on plant leaves is related to the surface properties of both pesticide and plant leaves, and the chemical composition of pesticides and the roughness and hydrophobicity of leaf surface are most important factors.^[^ ^59^ ^]^ Furthermore, the application of HLDP could increase the spread area of protectant droplets on mango leaves (Figure 2b). The leaf is primarily composed of trichomes and waxy layer to exhibit hydrophobic characteristics, which usually leads to pesticide drift.^[^ ^60^ ^]^ In the current study, the retention of protectants on mango leaves increased with the aid of HLDP, and that of HLDP‐CS complex was the highest (22.27 ± 0.59 mg cm^−2^) among various HLDP nano‐protectants (*F*_7,16_ = 164.9, *P *< 0.0001) (Figure 2c). The possible reason might be that the HLDP could decrease the surface tension of protectant droplets and promote their diffusion and adhesion, thus increasing the retention on plant leaves. Importantly, the plant uptake of all HLDP‐loaded protectants improved, and that of CS significantly increased by 4.85 times with the aid of HLDP (Figure 2d). The uptake efficiency of AIs mainly depends on their chemical properties, and the introduction of nanotechnology can improve the physicochemical characteristic of AIs to improve the delivery for enhanced bioactivity.^[^ ^28^, ^50^, ^61^ ^]^

### 2.4 High Direct Fungicidal Activity of HLDP Nano‐Protectants via Damaging the Cell Wall/Membrane

**Figure 3:**
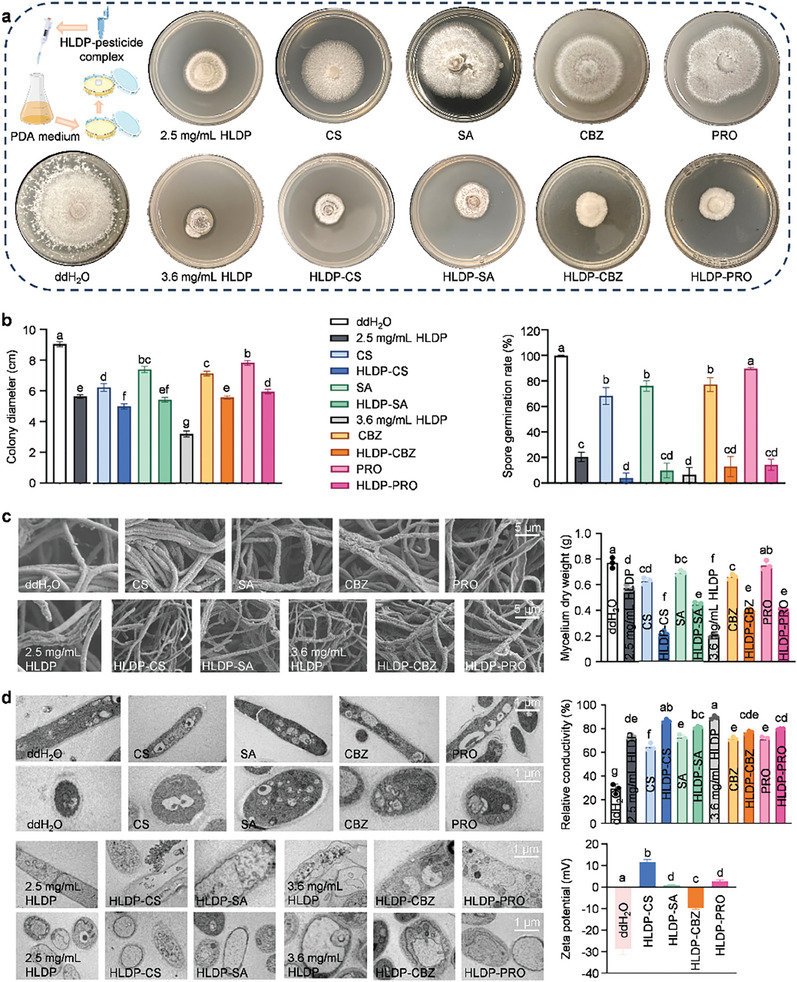
Direct fungicidal activities of HLDP nano‐protectants. a,b) Colony diameters of *C. gloeosporioides* inoculated on PDA medium containing HLDP nano‐protectants (protectant concentration: 5 mg mL^−1^) for 14 d. The data were recorded from three independent samples. Spore germination rates of *C. gloeosporioides* incubated with HLDP nano‐protectants (protectant concentration: 5 mg mL^−1^). The data were calculated from five independent samples at 12 h after the incubation. The ddH_2_O and HLDP were also tested. The concentration of HLDP was 2.5 mg mL^−1^ in HLDP‐CS and HLDP‐SA complexes, and it was 3.6 mg mL^−1^ in HLDP‐CBZ and HLDP‐PRO complexes. Different letters indicate significant differences according to the Brown‐Forsythe test (*P* < 0.05). c) Biological SEM image and dry weight of *C. gloeosporioides* mycelium treated with HLDP nano‐protectants (protectant concentration: 5 mg mL^−1^). Each treatment included three independent samples. d) TEM image and conductivity of mycelium treated with HLDP nano‐protectants (protectant concentration: 5 mg mL^−1^). Each treatment contained three independent samples. Zeta potential of mycelium treated with HLDP nano‐protectants (protectant concentration: 5 mg mL^−1^). Each treatment contained three independent samples.

The *C. gloeosporioides* strain on potato dextrose agar (PDA) medium was first employed to evaluate the direct fungicidal activities of HLDP nano‐protectants. Compared to the treatment of protectant alone, the colony diameters reduced from 6.2 ± 0.3 to 5.0 ± 0.2 cm for HLDP‐CS complex, from 7.4 ± 0.2 to 5.4 ± 0.2 cm for HLDP‐SA complex, from 7.1 ± 0.2 to 5.6 ± 0.1 cm for HLDP‐CBZ complex, and from 7.8 ± 0.2 to 6.0 ± 0.2 cm for HLDP‐PRO complex, respectively (*F*_10,22_ = 258.5, *P *< 0.0001) (**Figure** **3**a,b). In addition, the spore germination rates of *C. gloeosporioides* incubated with protectants remarkably reduced to 4.0 ± 2.9% (CS), 9.8 ± 6.4% (SA), 13.0 ± 9.6% (CBZ), and 14.4 ± 4.0% (PRO) with the aid of HLDP (*F*_10,44_ = 297.1, *P *< 0.0001). Notably, the concentration of HLDP in HLDP‐CS and HLDP‐SA complexes was 2.5 mg mL^−1^, and that in HLDP‐CBZ and HLDP‐PRO complexes was 3.6 mg mL^−1^. The HLDP alone at both concentrations could also inhibit the mycelial growth and spore germination. Thus, our results indicated that the direct fungicidal activities of HLDP nano‐protectants were mainly derived from HLDP, and the HLDP could be applied as an AI to effectively control *C. gloeosporioides*. The control effects of CBZ and PRO on mango anthracnose were not ideal possibly due to the application concentration and time.^[^ ^62^, ^63^, ^64^ ^]^ Similarly, a previous publication has reported that several types of nanomaterials such as mesoporous silica nanosphere, mesoporous Fe_3_O_4_ nanosphere, carbon quantum dot, polylactic acid‐glycolic copolymer, etc. can also suppress the spore germination and mycelial growth of *Verticillium dahliae*.^[^ ^65^ ^]^

The biological scanning electron microscope (SEM) was employed to observe the morphologies of mycelia treated with HLDP nano‐protectants (Figure 3c). Healthy *C. gloeosporioides* mycelia was linear, regular, uniform and smooth, whereas the mycelia treated with protectant alone turned to deformation and contraction. Impressively, the mycelial morphology and ultrastructure alternation caused by HLDP nano‐protectants were the markedly shriveled or flatted empty hyphae, and this phenomenon was similar to a previous study.^[^ ^28^ ^]^ After the treatment, the dry weights of mycelia decreased to 0.22 ± 0.02 g for HLDP‐CS complex, 0.44 ± 0.02 g for HLDP‐SA complex, 0.40 ± 0.01 g for HLDP‐CBZ complex and 0.39 ± 0.03 g for HLDP‐PRO complex, respectively (*F*_10,22_ = 191.2, *P *< 0.0001). Meanwhile, the TEM images showed that the healthy mycelia had intact cell walls, uniform plasma membranes, rich cytoplasmic matrix, and complete cell organelles, and cell wall was thick with the clear boundary between plasma membranes (Figure 3d). After the treatment with HLDP nano‐protectants, the cell structures were obviously destroyed, and the typical phenotypes included the damaged cell wall, blurred plasma membrane, and larger vacuoles.

The membrane permeability of mycelia treated with HLDP nano‐protectants was also examined, and the results revealed that the HLDP nano‐protectants could significantly increase the mycelia conductivity (Figure 3d). Thus, the treatment with HLDP nano‐protectants destroyed the integrity of cell wall/membrane, causing the leakage of cellular components and inhibiting the mycelial growth.^[^ ^66^ ^]^ The mycelial cells exhibited elongated and rounded shapes due to the different growth cycles. For the surface charge analysis, the zeta potential of *C. gloeosporioides* was −28.57 ± 2.8 mV, and those of *C. gloeosporioides* incubated with HLDP nano‐protectants were from −9.67 to 11.53 mV, which suggested that the interaction between *C. gloeosporioides* and HLDP nano‐protectants was electrostatic action. The electrostatic interaction is the most common mechanism for the interaction of microorganisms with nanomaterials, and the cationic nanomaterials can generate electrostatic attraction to negatively‐charged surface of microorganisms. Furthermore, the binding ability and bactericidal activity of nano‐pesticides are usually stronger when their surface carries more positive charges.^[^ ^67^, ^68^ ^]^ Based on above results, the HLDP‐CS nano‐protectant displayed the highest fungicidal activity and showed a great potential in actual production, which was employed to perform the following experiments.

### 2.5 Adverse Impacts of HLDP‐CS Nano‐Protectant on the Metabolism and Pathogenicity of *C. gloeosporioides*

**Figure 4:**
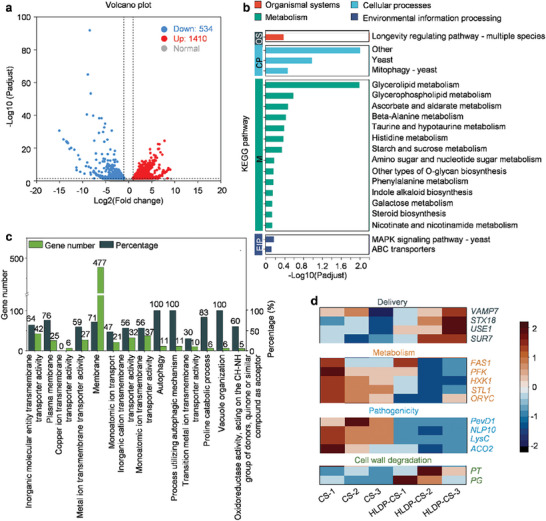
RNA‐seq analysis to illustrate the potential inhibitory mechanism of HLDP‐CS nano‐protectant. a) Analysis of DEGs in *C. gloeosporioides* treated with HLDP‐CS nano‐protectant and CS alone with the volcano plot. Up‐ and down‐regulated genes are represented by red and blue dots, respectively. b,c) KEGG and GO enrichments of DEGs. d) Heatmaps of various crucial genes related to direct fungicidal activity of HLDP‐CS nano‐protectant. Highly and lowly expressed genes are labeled as red and blue, respectively.

To further explore the potential inhibitory mechanism of HLDP‐CS nano‐protectant, RNA‐seq analysis was performed to examine the DEGs in mycelia treated with HLDP‐CS nano‐protectant and CS alone. The sequencing quality and correlation within biological replicates were high within each group (Table S1 and Figure S7, Supporting Information). RNA‐seq results revealed that a total of 1410 up‐ and 534 down‐regulated DEGs were identified in the mycelia treated with HLDP‐CS nano‐protectant (**Figure** **4**a). The enriched KEGG and GO terms were mainly related to biological process, transmembrane transporter activity, glycerolipid metabolism, intrinsic component of membrane, etc. (Figure 4b,c).

Compared to CS alone, the application of HLDP‐CS nano‐protectant up‐regulated some crucial genes related with delivery, including the members of vesicle‐associated membrane protein (VAMP) family (*VAMP7*), protein involved in retrograde vesicular transport *(STX18*) and transmembrane protein (*SUR7*) (Figure 4d). *VAMP7* has been reported to be involved in a variety of membrane trafficking events.^[^ ^69^ ^]^ *STX18* transports between the Golgi apparatus and endoplasmic reticulum.^[^ ^70^ ^]^ *SUR7* localizes to MCC/eisosomes, which is the plasma membrane to maintain proper cell wall morphogenesis.^[^ ^71^ ^]^ Up‐regulation of these genes suggested that the HLDP‐CS nano‐protectant could accelerate the translocation of exogenous substances across the cell membrane, which might destroy the integrity of cell membrane. In addition, the up‐regulation of *PT* (polygalacturonase) and *PG* (pectinase) also indicated that the structure of cell wall was damaged after the treatment with HLDP‐CS nano‐protectant, which suppressed the mycelial growth and spread.

Our results demonstrated that the genes involved in metabolism pathway, such as *FAS1* (microbial type I fatty acid synthases), *HXK1* (N‐acetylglucosamine kinase), *STL1* (a member of the sugar transporter family) and *ORYC* (MFS‐type transporter oryC) were down‐regulated in the mycelia treated with HLDP‐CS nano‐protectant, indicating that HLDP‐CS nano‐protectant could inhibit the mycelial metabolism.^[^ ^72^, ^73^, ^74^ ^]^ Furthermore, as positive regulators in the pathogenicity, *PevD1* (Phyllobacterium endophyticum virulence factor D1), *NLP10* (NOD‐Like Protein 10), *LysC* (Lysine‐specific protease C) and *ACO2* (Aconitase 2) were all down‐regulated in the mycelia treated with HLDP‐CS nano‐protectant, suggesting the reduced pathogenicity.^[^ ^75^, ^76^, ^77^, ^78^ ^]^ The qRT‐PCR results also supported the transcriptome data (Figure S8, Supporting Information). Therefore, the direct fungicidal activity of HLDP‐CS nano‐protectant might be derived from the membrane damage, mycelial metabolism inhibition and pathogenicity reduction.

### 2.6 Excellent Protective Effects of HLDP‐CS Nano‐Protectant on Mango Fruits toward Anthracnose

**Figure 5:**
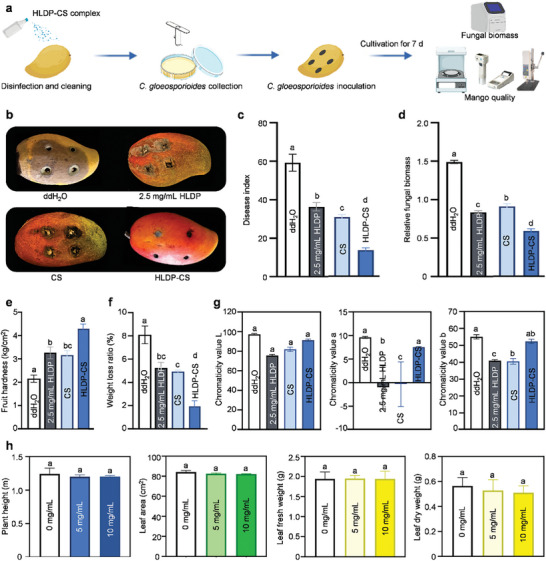
Protective effects of HLDP‐CS nano‐protectant on mango fruits toward anthracnose. a) Experimental flow diagram. b–d) Control effects of HLDP‐CS nano‐protectant on *C. gloeosporioides*. The mango fruits sprayed with HLDP‐CS nano‐protectant (CS concentration: 5 mg mL^−1^) were inoculated with *C. gloeosporioides*. The disease index was calculated at 7 d after the inoculation. Each group contained nine fruits, which was repeated three times. Fungal biomass was quantified, and the expression level of *actin* gene (*ACT3*) was normalized to the abundance of mango housekeeping gene *LOC123201910*. Each treatment comprised three independent samples. Different letters indicate significant differences according to the Brown‐Forsythe test (*P* < 0.05). e–g) Quality of mango fruits treated with HLDP‐CS nano‐protectant. Each treatment consisted of nine fruits with three replicates. h) Potential adverse impacts of HLDP‐CS nano‐protectant on mango seedlings. The mango seedlings were sprayed with HLDP‐CS nano‐protectant (CS concentration: 5 and 10 mg mL^−1^). The height of seedlings, leaf area, and fresh and dry weight of leaves were recorded at 14 d after the treatment. Each treatment included three independent samples.

The HLDP‐CS nano‐protectant was sprayed onto mango fruits, and *C. gloeosporioides* was then inoculated to examine the protective effects (**Figure** **5**a). As depicted in Figure 5b, the infection symptoms of fruits treated with HLDP‐CS nano‐protectant were the mildest among various formulations. The disease index of fruits treated with HLDP‐CS nano‐protectant was 13.58 ± 1.2, which was significantly lower than those treated with ddH_2_O (59.26 ± 4.5), 2.5 mg mL^−1^ HLDP (36.39 ± 2.2) and CS (30.86 ± 1.2) (*F*_3,8_ = 154.6, *P *< 0.0001) (Figure 5c). Additionally, the relative fungal biomass of mycelia treated with HLDP‐CS nano‐protectant decreased to 0.59, revealing its excellent protective effects (Figure 5d).

The hardness, weight loss and color of mango fruits treated with HLDP‐CS nano‐protectant were also examined to evaluate the fruit quality. As shown in Figure 5e,f, the fruits treated with HLDP‐CS nano‐protectant were the hardest (4.3 kg cm^−2^), and their weight loss ratio was the lowest (1.92%) among various formulations. The chromaticity value L represents the brightness of fruit color, and value a and b represent the degrees of green to red peel, and blue to yellow peel, respectively.^[^ ^79^ ^]^ Our results indicated that the application of HLDP‐CS nano‐protectant could promote the yellowing and reddening of peel (Figure 5g). Chitosan in the form of nanoemulsions is more effective as a biofungicide for controlling anthracnose and alleviating the adverse impacts on the quality of fresh fruits.^[^ ^80^ ^]^ The potential adverse impacts of HLDP‐CS nano‐protectant on mango seedlings were also evaluated, and our results revealed that its application exhibited no significant impacts on seedling growth at both working concentration (CS concentration: 5 mg mL^−1^) and high concentration (CS concentration: 10 mg mL^−1^) (Figure 5h). Furthermore, no residue of HLDP‐CS nano‐protectant was tested in mango leaves and fruits (Figure S9, Supporting Information). Low residues indicate a reduced risk of accidental environmental exposure, leading to a wider adoption of nano‐protectants in agricultural applications.^[^ ^81^, ^82^ ^]^ However, a previous study reports that 5 mg mL^−1^ HLDP‐SA nano‐protectant can inhibit the germination and growth of cotton and rice seeds, and the possible reason might be that the nanoparticles can penetrate through the seed coat to result in the toxic effects.^[^ ^28^, ^83^ ^]^ In terms of scalability and economic feasibility, the current synthetic cost of HLDP is as low as 14 g per $ in the laboratory. The application cost of HLDP‐CS nano‐protectant can be further decreased for mass production in factories.

### 2.7 Amplified Plant Immune Responses Induced by HLDP‐CS Nano‐Protectant via Activating the Biosynthesis of Secondary Metabolites and Plant Hormones

**Figure 6:**
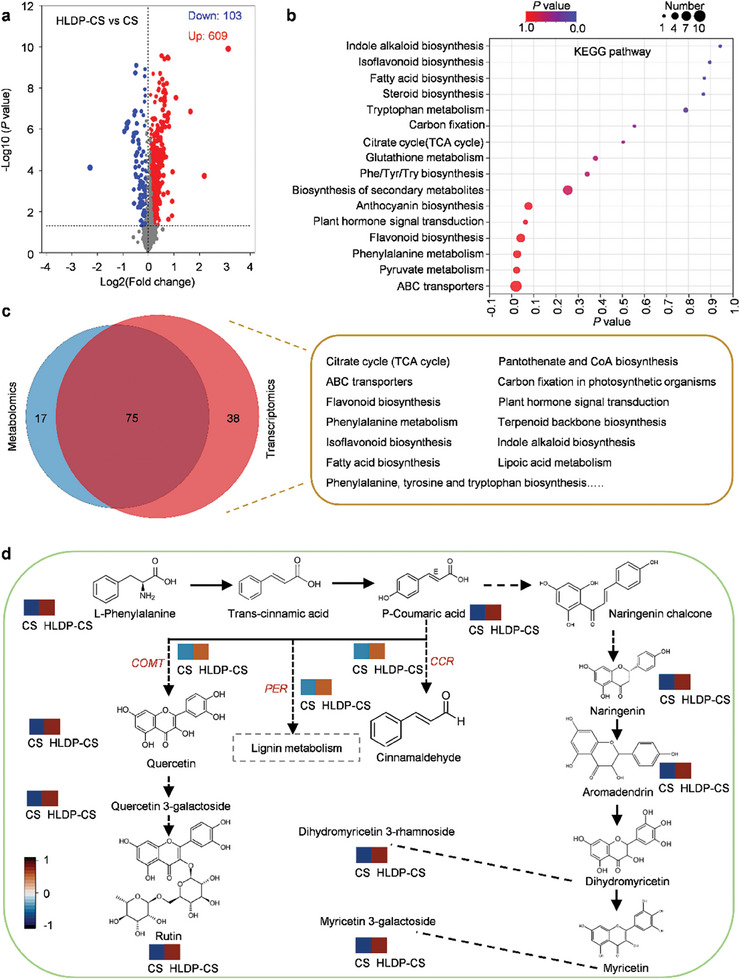
Conjoint analysis of metabonomics and transcriptomics for amplified plant immune responses induced by HLDP‐CS nano‐protectant. a) Analysis of DEMs in mango fruits treated with HLDP‐CS nano‐protectant and CS alone with the volcano plot. Up‐ and down‐regulated genes are represented by red and blue dots, respectively. b) KEGG enrichment of DEMs. c) Common pathways in the metabonomics and transcriptomics. Typical common pathways are provided. d) Effects of HLDP‐CS nano‐protectant on plant secondary metabolite signaling pathway. The amounts of DEMs are indicated by the heatmap using Log2(Fold change). Up‐regulated DEGs are marked with red. *COMT*: Catechol‐O‐methyltransferase; PER: Phenylalanine ammonia‐lyase; *CCR*: Cinnamoyl‐CoA reductase.

To explore the mechanism of amplified plant immune responses, the metabolome sequencing was carried out to analyze the DEMs in mango fruits treated with HLDP‐CS nano‐protectant and CS alone. Principal component analysis (PCA) was used to test the correlation of replicate samples, and two principal components accounted for 40.1% and 28.3% of the variance, indicating good and reliable experimental reproducibility (Figure S10, Supporting Information). As shown in **Figure** **6**a, the 609 up‐ and 103 down‐regulated DEMs were identified in the fruits treated with HLDP‐CS nano‐protectant. KEGG enrichment indicated that the DEMs were primarily enriched in the biosynthesis of secondary metabolites, plant hormone signal transduction, steroid biosynthesis, isoflavonoid biosynthesis, etc., which indicated that the HLDP‐CS nano‐protectant activated the plant immune responses via regulating the synthesis of secondary metabolites and plant hormones (Figure 6b). The high levels of trans‐cinnamic acid, quercetin, rutin and pentoxifylline suggested that the production of secondary metabolites was induced in the fruits treated with HLDP‐CS nano‐protectant (Figure 6d; Figure S10, Supporting Information). Similarly, the up‐regulated DEMs also included SA and ABA (abscisic acid), suggesting that HLDP‐CS nano‐protectant could activate the production of endogenous plant hormones to amplify the plant immune responses (Figure S11, Supporting Information).

The treated mango fruits were also harvested for RNA‐seq, and the sequencing quality and correlation within biological replicates were good for the following analysis (Table S2 and Figure S12a, Supporting Information). Compared to CS alone, a total of 2779 DEGs were detected, which were classified into several categories (Figure S12b,c, Supporting Information). The genes related with secondary metabolism, metabolism and plant defence were up‐regulated in the fruits treated with HLDP‐CS nano‐protectant (Figure S13, Supporting Information). The conjoint analysis of metabolomics and transcriptomics was carried out to identify 75 common pathways, including citrate cycle (TCA cycle), plant hormone signal transduction, flavonoid biosynthesis, isoflavonoid biosynthesis, phenylalanine, tyrosine and tryptophan biosynthesis, etc. (Figure 6c).

Previous publications report that the amounts of flavonoid, anthocyanidin and tannin significantly increase in response to the inoculation of *C. gloeosporioides*.^[^ ^84^, ^85^ ^]^ Our transcriptomic and metabolome data suggested that many DEGs and DEMs were enriched in the biosynthesis of secondary metabolites, which has been shown to play important roles in plant immune responses. The conjoint analysis revealed that three genes were correlated to the production of nine metabolites in the phenylpropanoid and flavonoid pathway (Figure 6d). The genes included *COMT* (caffeic acid 3‐O‐methyltransferase), PER (phenylalanine ammonia‐lyase) and *CCR* (cinnamoyl‐CoA reductase), and the metabolites included L‐Phenylalanine, quercetin, quercetin 3‐galactoside, rutin, P‐coumaric acid, naringenin, aromadendrin, dihydromyricetin 3‐rhamnoside and myricetin 3‐galactoside. The HPLC was further carried out to verify the metabolome data, and the contents of gallic acid and rutin in the fruits treated with HLDP‐CS nano‐protectant significantly increased by 1.69 and 1.55 times those treated with CS alone, respectively (Figure S14, Supporting Information). As an important secondary metabolite, rutin plays an important role in the process of disease resistance, which helps plants cope with biological stress and pathogen infection.^[^ ^86^ ^]^ For instance, the external application of rutin can inhibit the proliferation of *Xanthomonas oryzae* pv*. oryzae* and improve the resistance of rice.^[^ ^87^ ^]^

Plant hormones play a crucial role in the growth and defence against pathogens.^[^ ^88^ ^]^ Our results suggested that the synthesis and signal transduction of ABA, gibberellin (GA) and ethylene were activated in the fruits treated with HLDP‐CS nano‐protectant, and their activation had positive effects on plant physiological processes and disease resistance (Figure S15, Supporting Information). More specifically, ethylene binds with its receptor to activate the ethylene signaling pathway, which can improve the plant resistance against pathogen infection.^[^ ^89^ ^]^ Similarly, GAs can promote the growth and development of plants while simultaneously influencing the plant immune responses.^[^ ^90^ ^]^ ABA is regarded to be involved in responding to various biological stresses.^[^ ^91^, ^92^ ^]^ Overall, the protective effects of HLDP‐CS nano‐protectant on mango fruits are derived from both direct fungicidal activity of HLDP and plant immune response induced by HLDP‐loaded CS. A similar publication reports that the HLDP can assemble with SA to form a stable complex, which amplifies the plant defense induced by SA.^[^ ^28^ ^]^

## 3 Conclusion

In this work, we designed and synthesized HLDP nanomaterial to prepare a series of HLDP nano‐protectants based on plant elicitors and fungicides, and explored the best nano‐protectant for anthracnose management. The self‐assembled HLDP‐CS nano‐protectant displayed the best control effects on mango anthracnose via the direct pathogen inhibition and amplified plant immune responses (Figure S16, Supporting Information). The complexation of HLDP with CS was achieved via hydrophobic interaction to form nanoscale spherical particles, which remarkably improved the deposition and adhesion of CS droplets on mango leaves and the plant uptake of HLDP‐loaded CS. On one hand, the HLDP could be applied as an AI to directly interact with mycelium via electrostatic interaction to remarkably damage the cell wall/membrane, which suppressed the spore germination and mycelial growth. The transcriptome results also supported that HLDP‐CS nano‐protectant could inhibit the mycelial metabolism and reduce the pathogenicity. On the other hand, HLDP could be applied as an adjuvant for CS to amplify the plant immune responses via accelerating the biosynthesis of secondary metabolites and plant hormones. The HLDP‐CS protectant displayed the excellent protective effects on mango fruits, and achieved fruit quality improvement. Overall, the current study comprehensively displayed the multiple missions of nanomaterial HLDP in the preparation of a self‐assembled nano‐protectant, which could be applied to achieve high‐efficiency green control of anthracnose.

## 4 Experimental Section

### Chemical Reagents

The standards of CS, SA, CBZ (97%) and PRO (99%) were purchased from Sigma‐Aldrich (USA). The ε‐caprolactone (ε‐CL), Sn(Oct)_2_, 1‐butanol, 2‐bromo‐2‐methylpropionyl bromide (BIBB), and trimethylamine (TEA, 99%) purchased from Heowns BioChem Technologies (China), 2‐(Dimethyl amino) ethyl methacrylate (DMAEMA, 99%) purchased from Energy Chemical (China), and N,N,N′,N′,N″‐Pentamethyl diethylenetriamine (PMDETA, 98%) and CuBr (99.999%) purchased from Sigma‐Aldrich (USA) were used for HLDP synthesis. Dichloromethane (DCM) and other chemical reagents were obtained from Beijing Chemical Works (China).

### HLDP Synthesis

The HLDP was synthesized according to the procedure described by Yin et al.^[^ ^28^ ^]^ Initially, 1‐butanol (1.35 mmol), ε‐CL (54 mmol) and Sn(Oct)_2_ (310 mg) were incubated in an oil bath at 90 °C for 9 h. Subsequently, the mixture was added with DCM (30 mL), and the mixture was precipitated by adding cold methanol to obtain the PCL. BIBB (13.04 mmol), THF (30 mL) and TEA (40 mmol) were then added dropwise to PCL solution (0.58 mmol). The reaction was terminated by adding methanol, and the precipitate was collected by filtration to obtain the white powder of PCL‐Br. The polymerization reaction was carried out using PCL‐Br (0.04 mmol), DMAEMA (6.07 mmol), THF (2 mL), CuBr (0.1 mmol), and PMDETA (0.21 mmol) at 65 °C. After the reaction, the reaction system was treated with liquid nitrogen, and then warmed to room temperature. HLDP was obtained as the white powder after the dialysis in water and freeze‐drying.

### Loading Efficiency Measurement of HLDP toward Various Protectants

The standards of CS, SA, CBZ, and PRO were used to prepare a series of dilutions, and their contents were measured using the HPLC to construct the standard calibration curves. The detection conditions for SA, CBZ, and PRO, as well as their HLDP nano‐protectants, included: C18 column, mobile phase of acetonitrile‐0.1% acetic acid aqueous solution (v/v: 90/10), detection wavelength of 254 nm, flow rate of 1 mL min^−1^, injection volume of 20 µL, and column temperature of 30 °C. CS was acetylated and acid hydrolyzed into glucosamine salts to calculate the content of CS. The detection conditions for glucosamine salt included: amino column, mobile phase of acetonitrile‐water (v/v: 80/20), flow rate of 1 mL min^−1^, injection volume of 20 µL, and column temperature of 30 °C.

The HLDP solution (5 mg mL^−1^) was incubated with the solutions of CS, SA, CBZ, and PRO, respectively at the mass ratio of 1:1 for 30 min to prepare various HLDP nano‐protectants. Subsequently, various solutions of HLDP nano‐protectants were dialyzed in ddH_2_O using the dialysis bag with the molecular weight cut‐off of 1000 Da (Shanghai Yuanye Biotechnology Co., Ltd., China) for 24 h. The protectant content inside the dialysis bag was determined by HPLC. The protectant loading efficiency was calculated using the equation of protectant loading efficiency (%) = weight of protectant loaded in nano‐complex ÷ weight of nano‐complex × 100. A series of HLDP nano‐protectants were prepared according to the corresponding protectant loading efficiencies in the following experiments.

### ITC Assay of HLDP with Various Protectants

The dominant interaction force of protectant with HLDP was analyzed using the ITC method. The 1000 µL of HLDP aqueous solution (0.138 mm) was titrated with 250 µL protectant aqueous solution (1 mm) in Nano ITC (TA Instruments Waters, USA). During each injection, the heating temperature of interaction was calculated by integrating each titration peak via the Origin7 software (OriginLab Co., USA). The test temperature was 25 °C, and ΔG value was calculated using the formula of ΔG = ΔH – TΔS.

### Particle Size Measurement and Morphology Observation of HLDP Nano‐Protectants

The solutions of HLDP nano‐protectants (HLDP concentration: 5 mg mL^−1^) were prepared according to the corresponding protectant loading efficiencies as described above. Their particle sizes and zeta‐potentials were measured by the dynamic light scattering (DLS) with a Zeta sizer Nano Series (Malvern Instruments, UK) at 25 °C. The HLDP and protectant alone were also tested. Each treatment contained three independent samples. Meanwhile, the morphologies of above samples were examined using the TEM (JEOL‐2100F, Japan). Each sample was dropped on the microgrid and treated with 2% phosphotungstic acid for observation.

### Photostability Test of HLDP Nano‐Protectants

The 10 mL solutions of HLDP nano‐protectants (protectant concentration: 5 mg mL^−1^) and protectant alone were individually placed in quartz test tubes, and treated in the 365 nm photocatalytic reaction chamber (300 W high‐pressure mercury lamp, XPA‐7, China). The retention ratio of each protectant was analyzed using the HPLC at 0, 3, 6, 12, and 24 h after the irradiation. Each treatment contained three independent samples.

### Dissolution Test of HLDP Nano‐Protectants

HLDP nano‐protectants (protectant concentration: 5 mg mL^−1^) were dissolved in ultrapure deionized water under oscillation conditions, collected at 1, 2, and 3 d under oscillation conditions, and used to determine the particle size, following the above method. Each treatment included three independent samples.

### Stability Text of HLDP Nano‐Protectants

Zeta‐potentials and particle sizes of HLDP nano‐protectants (protectant concentration: 5 mg mL^−1^) were measured at 1, 3, and 7 d after the storage at room temperature, following the above method. Each treatment included three independent samples.

### Contact Angle and Retention Measurement of HLDP Nano‐Protectants on Mango Leaves

The solutions of HLDP nano‐protectants were used to test the contact angle and retention on mango leaves (*Mangifera indica* L.). For contact angle analysis, the 5 µL solutions of HLDP nano‐protectants (protectant concentration: 5 mg mL^−1^) and protectant alone were vertically dripped onto mango leaf surface using Optical Contact Angle Meter (Date Physics OCA50AF, Germany). The contact angle was analyzed by elliptical fitting algorithm. The test was repeated three times for each solution. For retention analysis, the mango leaves (0.8 cm^2^) were weighted, and then completely immersed in above solutions for 30 s. The treated leaves were taken out and weighed until no droplets fell. The retention was calculated using the equation of retention (mg cm^−2^) = (leaf weight after immersion – leaf weight before immersion) ÷ leaf area. The experiment was performed eight times.

### Plant Uptake Analysis of HLDP Nano‐Protectants

Fresh mango fruits were immersed in the solutions of HLDP nano‐protectants (protectant concentration: 5 mg mL^−1^) and protectant alone for 10 min, air‐dried, and then cleaned with ddH_2_O at 6 h after the immersion. The SA, CS, CBZ, and PRO were extracted from the homogenized fruit (5 g) using 20 mL of acetonitrile acetate. After the centrifugation, the supernatant (10 mL) was evaporated with the gentle stream of nitrogen (40 °C) until the volume was reduced to 1 mL.^[^ ^28^ ^]^ Each sample was purified using the polytetrafluoroethylene membrane (Haiming Zhongli Filtering Equipment Factory, China) for liquid chromatography‐tandem mass spectrometry (LC−MS/MS) analysis on the ACQUITY UPLC‐TQD system equipped with the ACQUITY UPLC BEH C18 column and amino column from Waters Corporation (USA). The separation of the analytes was achieved using the mobile phase composed of water and acetonitrile (1:9 v/v) at the flow rate of 400 µL min^−1^. The injection volume was 20 µL, and the column temperature was set at 30 °C. Other conditions included the capillary voltage of 4,000 V, heater block temperature of 400 °C, and interface temperature of 300 °C. Each sample was tested three times.

### Direct Fungicidal Activity Assay of HLDP Nano‐Protectants toward *C. gloeosporioides*

The spore suspension of *C. gloeosporioides* strain was adjusted to 2 × 10^7^ cfu mL^−1^, which was cultured on PDA medium under dark condition at 28 °C. The colonies (5 mm) were then inoculated on PDA medium containing HLDP nano‐protectants (protectant concentration: 5 mg mL^−1^) and protectant alone, respectively. The ddH_2_O and HLDP (2.5 mg mL^−1^ HLDP: HLDP concentration in HLDP‐CS and HLDP‐SA complexes; 3.6 mg mL^−1^ HLDP: HLDP concentration in HLDP‐CBZ and HLDP‐PRO complexes) were also tested. The colony diameter was recorded at 14 d after the inoculation, and each treatment was repeated three times. The spore suspension (200 µL) was incubated with the same volume of HLDP nano‐protectants (protectant concentration: 5 mg mL^−1^) and protectant alone, respectively. The ddH_2_O and HLDP (2.5 and 3.6 mg mL^−1^) were also tested. The spore germination rate was calculated at 12 h after the incubation. The test was repeated five times for each treatment.

### Mycelial Growth Assay of *C. gloeosporioides* Treated with HLDP Nano‐Protectants

According to the method described by Yang et al.,^[^ ^93^ ^]^ the biological SEM was employed to observe the morphologies of mycelia treated with HLDP nano‐protectants. In brief, the *C. gloeosporioides* strain was treated with various formulations as described above, and the PDA samples were harvested at 14 d after the treatment, soaked in the fixative solution (glutaraldehyde, 2.5%, pH = 7.2–7.4) for 24 h, sputter‐coated with gold, and photographed using the SEM (HITACHI SU8010, Japan). For TEM observation, the PDA samples were promptly placed into glutaraldehyde for 24 h, and then washed with 0.1 mol L^−1^ PBS (pH = 7.4). The samples were then fixed with 1% OsO_4_ for 2 h, washed with PBS to remove OsO_4_, and dehydrated with ethanol. Following infiltration and embedding the cell pellet, ultrathin sections were cut with the ultramicrotome. The samples were observed and photographed using the TEM.

The dry weight, relative conductivity and zeta potential of mycelia treated with HLDP nano‐protectants were examined in the following experiments. The 1 mL of spore suspension (2 × 10^7^ cfu mL^−1^) was added and cultured in 90 mL potato dextrose broth (PDB) liquid medium at 28 °C for 5 d. For dry weight measurement, the mycelium was harvested, suspended in PDB medium containing HLDP nano‐protectants (protectant concentration: 5 mg mL^−1^) and protectant alone, and cultured at 28 °C for 60 h. The ddH_2_O and HLDP (2.5 and 3.6 mg mL^−1^) were also tested. The mycelium was then collected, washed, dried at 80 °C, and then weighed. Each treatment was replicated three times. For conductivity measurement, the mycelium was suspended in the formulations of HLDP nano‐protectants (protectant concentration: 5 mg mL^−1^) and protectant alone, respectively. The ddH_2_O and HLDP (2.5 and 3.6 mg mL^−1^) were also tested. The conductivity was measured using the conductivity meter (DDS‐307, China). The mycelium was boiled for 5 min, and the conductivity was measured again. Each treatment included three independent samples. The formula of relative conductivity (%) = conductivity of mycelium before boiling ÷ conductivity of mycelium after boiling × 100 was employed to calculate the relative conductivity. For zeta potential measurement, the mycelium was suspended in the formulations of HLDP nano‐protectants (protectant concentration: 5 mg mL^−1^) and protectant alone, respectively. The ddH_2_O and HLDP (2.5 and 3.6 mg mL^−1^) were also tested. The zeta potentials of above samples were determined using the Zetasizer Nano ZS (Malvern Instruments Co., UK) at 25 °C. The test was repeated three times for each treatment.

### RNA‐seq and qRT‐PCR for Analyzing the Direct Fungicidal Activity of HLDP‐CS Nano‐Protectant

The *C. gloeosporioides* was cultured on PDA medium containing HLDP‐CS nano‐protectant (protectant concentration: 5 mg mL^−1^) and CS alone for 14 d, and harvested for RNA extraction. Concurrently, above samples were treated with RNase A (1 µg mL^−1^) at 37 °C for 15 min, and the total RNA was extracted using the EasyPure Viral DNA/RNA Kit (China) and reverse transcribed using the PrimeScriptTM RT reagent Kit (Japan) to obtain the cDNA. Each treatment comprised three independent samples. The samples were amplified, and the PCR products were purified to obtain the final sequencing library. The Illumina Novaseq 6000 high‐throughput sequencing platform (Illumina, USA) was used for sequencing. *C. gloeosporioides* (GCA_02 143 2615.1) reference from NCBI (https://www.ncbi.nlm.nih.gov/) was adopted to achieve the sequence alignment of clean paired‐end reads. DESeq was employed to analyze the DEGs, and FDR < 0.01 and Log2(fold change) ≥ 1.0 were the screen conditions. Gene ontology (GO) and Kyoto Encyclopedia of Genes and Genomes (KEGG) enrichment analysis were carried out for gene enrichment and functional annotation.

The above RNA samples were reverse transcribed using the ReverTra Ace QPCR RT Master Mix with gDNA Remover kit (Toyobo, FSQ‐301, China). The expression levels of target genes were further verified by qRT‐PCR. Primers are shown in Table S3 (Supporting Information). The *GAPDH* was employed as the reference gene, and the expression level of each target gene was determined using the 2^−∆∆CT^ methods.^[^ ^94^ ^]^ Reactions were performed in triplicate under the following conditions: one cycle at 95 °C for 10 min, followed by 40 cycles at 95 °C for 15 s, 60 °C for 30 s, and 72 °C for 35 s. Each treatment comprised nine independent samples.

### Control Effect Assay of HLDP‐CS Nano‐Protectant toward Mango Anthracnose

The mango fruits were sprayed with the formulations of HLDP‐CS nano‐protectant (CS concentration: 5 mg mL^−1^, 10 mL per fruit) and CS alone, and the *C. gloeosporioides* colony was inoculated on the fruits at 24 h after the spraying under suitable growth conditions (28 °C, 16/8 h light/dark and 70% relative humidity). Each treatment included nine fruits, which was repeated three times. The disease index was recorded at 7 d after the inoculation. According to the previous method,^[^ ^95^ ^]^ the disease severity levels could be categorized as follows. Grade 0: No disease symptoms. Grade 1: Disease lesions occupy < 10% of the fruit surface area. Grade 3: Disease lesions occupy 10–20% of the fruit surface area. Grade 5: Disease lesions occupy 21–40% of the fruit surface area. Grade 7: Disease lesions occupy 41–60% of the fruit surface area. Grade 9: Disease lesions occupy > 60% of the fruit surface area. The disease index was calculated using the following equation.

(1) $$\begin{array}{c} \mathrm{Disease}\;\mathrm{index}=\frac{\sum \left(\mathrm{Number}\;\mathrm{of}\;\mathrm{fruits}\;\mathrm{in}\;\mathrm{each}\;\mathrm{severity}\;\mathrm{level}\times \mathrm{grade}\right)}{\mathrm{Number}\;\mathrm{of}\;\mathrm{total}\;\mathrm{surveyed}\;\mathrm{fruits}\times 9}\times 100\;\;\;\; \end{array}$$

Fungal biomass was also quantified in mango fruits to investigate the infection severity. Based on the previous method,^[^ ^96^ ^]^ the fungal biomass was examined using qRT‐PCR, and the expression level of *actin* gene (*ACT3*) was normalized to the abundance of mango housekeeping gene *LOC123201910*. Each treatment comprised three independent samples.

### Quality Assay of Mango Fruits Treated with HLDP‐CS Nano‐Protectant

To explore the sensory quality of mango fruits treated with HLDP‐CS nano‐protectant, the hardness, weight loss, and color of above treated fruits were examined in the current study. The hardness of above fruits was measured using the hardness tester (GY‐4, China). The hardness tester was perpendicular placed on the fruit surface, and the indenter of hardness tester was evenly pressed into the fruits to record the hardness. Weight loss was determined using the weighing method, and the fruit weight was measured before and after 7 d storage. The weight loss was calculated using the formula of weight loss (%) = (fruit weight before storage – fruit weight after storage) ÷ fruit weight before storage × 100. The color was assessed using the handheld colorimeter (CR400, Japan), and the focal length of colorimeter was adjusted and pointed at the fruit surface to record the color. Each treatment consisted of nine fruits, which was repeated three times.

To evaluate the potential adverse impacts of HLDP‐CS nano‐protectant on the growth of mango seedlings, the mango leaves were sprayed with HLDP‐CS nano‐protectant (CS concentration: 5 and 10 mg mL^−1^, 120 mL per seedling) and ddH_2_O, and the seedling height, leaf area, and the fresh and dry weight of leaves were recorded at 14 d after the spraying. The seedling height was calculated from three independent seedlings, and the leaf area and the fresh and dry weight of leaves were calculated from three independent leaves.

To evaluate the residue of HLDP‐CS protectant, CS contents were determined in mango leaves and fruits according to the <Determination of 450 pesticides and related chemicals residues in fruits and vegetables‐LC‐MS‐MS method> (GB/T 20769–2008). The mango leaves and fruits were sprayed with HLDP‐CS nano‐protectant (CS concentration: 5 mg mL^−1^, 20 mL per leaf/fruit), and the CS content was determined using the LC‐MS/MS at 7 d after the spraying. Each treatment was repeated three times.

### Metabolomics and RNA‐seq for Analyzing the Plant Immune Responses Induced by HLDP‐CS Nano‐Protectant

The solutions of HLDP‐CS nano‐protectant (CS concentration: 5 mg mL^−1^, 10 mL per fruit) and CS alone were sprayed on mango fruits, and *C. gloeosporioides* colony was then inoculated at 24 h after the spraying. Fruits were harvested, freeze‐dried, and grinded using the mixer mill (MW 400, Germany) at 7 d after the inoculation. The 100 mg sample powder was applied for LC‐MS/MS analysis, and the raw data was analyzed using the Progenesis QI software (Waters Corporation, USA). Subsequently, the metabolites with variable importance in the projection (VIP) > 1 and *P* < 0.05 were identified as the differential metabolites, determined by the OPLS‐DA model and *P*‐value generated by student's *t* test. The DEMs between two groups were then analyzed for their biochemical pathways using metabolic enrichment and pathway analysis based on the KEGG database (http://www.genome.jp/kegg/). Each treatment consisted of six independent samples.

Above treated mango fruits were harvested for RNA‐seq similarly as above experiment for fungus. Each treatment included three independent samples. The fruit samples were subjected to a combined analysis of transcriptomics and metabolomics.

### Content Measurement of Various Substances Related with Plant Immune Responses

The mango fruits were treated with HLDP‐CS nano‐protectant and CS alone, and then inoculated with *C. gloeosporioides* similarly as above. The fruits were harvested to measure the contents of total phenols and flavonoids at 7 d after the inoculation. For sample preparation, the 9 mL of pre‐cooled 1% HCl‐methanol solution was added with 1 g fruit tissue. The mixture was extracted and centrifuged at 8000 rpm, and the supernatant was collected for HPLC. Gallic acid was used to represent the total phenols, and its standard curve was constructed. The detection conditions for gallic acid: column C18, mobile phase of methanol and 0.05% phosphoric acid (5:95 v/v) solution, flow rate of 1 mL min^−1^, detection wavelength of 273 nm, injection volume of 10 µL, and column temperature of 30 °C. The representative flavonoid rutin was quantified according to the method described by Palafox‐Carlos et al.^[^ ^97^ ^]^ The HPLC conditions were as follows: column C18, mobile phase of methanol and 0.1% glacial acetic acid (32:68 v/v) solution, flow rate of 1 mL min^−1^, detection wavelength of 261 nm, injection volume of 10 µL, and column temperature of 30 °C. Each sample was tested three times.

### Data Analysis

The data were analyzed using the Prism 10 software (USA). Descriptive data were expressed as the mean and standard error. Significant difference between two or three groups was determined using the ratio paired *t*‐test or Brown‐Forsythe test at *P* < 0.05 significance level, respectively.

## Conflict of Interest

The authors declare no conflict of interest.

## Author Contributions

S.Y., X.S., and J.Y. designed the experiments. J.Y. performed most of the experiments. J.Z. performed plant transcriptome analysis. Z.W. and Z.F. performed ITC experiments. M.Y., H.C., J.S., J.L., and H.G. provided material resources. S.Y. and J.Y. wrote the manuscript. All authors edited the manuscript.

## Supporting information



Supporting Information

Supplemental Data 1

## Data Availability

The data that support the findings of this study are available from the corresponding author upon reasonable request.
