# Supplementary material for: Preparation of Multifunctional Nano‐Protectants for High‐Efficiency Green Control of Anthracnose
Source: Adv Sci (Weinh). 2024 Nov 18;11(48):2410585. doi: 10.1002/advs.202410585 (PMC11672290; doi:10.1002/advs.202410585)
Supplement: Supplementary file 1 — Supporting Information [file ADVS-11-2410585-s001.docx]

Supplementary information

Preparation of multifunctional nano-protectants for high-efficiency green control of anthracnose

***Jiaming Yin*^1,2^, *Jiajia Zhao*^1,2^, *Zeng Wang*^1^, *Zhen Fang*^2^, *Huiming Guo*^3,4^, *Hongmei Cheng*^3,4^, *Jie Li*^5^, *Jie Shen*^1,2^, *Meizhen Yin*^5^, *Xiaofeng Su*^3,4🖂^ and *Shuo Yan*^1,2🖂^**

^1^Frontiers Science Center for Molecular Design Breeding, Department of Plant Biosecurity, College of Plant Protection, China Agricultural University, Beijing, China. ^2^Sanya Institute of China Agricultural University, Sanya, China. ^3^National Key Laboratory of Agricultural Microbiology, Biotechnology Research Institute, Chinese Academy of Agricultural Sciences, Beijing, China. ^4^National Nanfan Research Institute, Chinese Academy of Agricultural Sciences, Sanya, China. ^5^State Key Laboratory of Chemical Resource Engineering, Beijing Lab of Biomedical Materials, Beijing University of Chemical Technology, Beijing, China.

🖂e-mail: suxiaofeng@caas.cn; yanshuo2011@foxmail.com

**
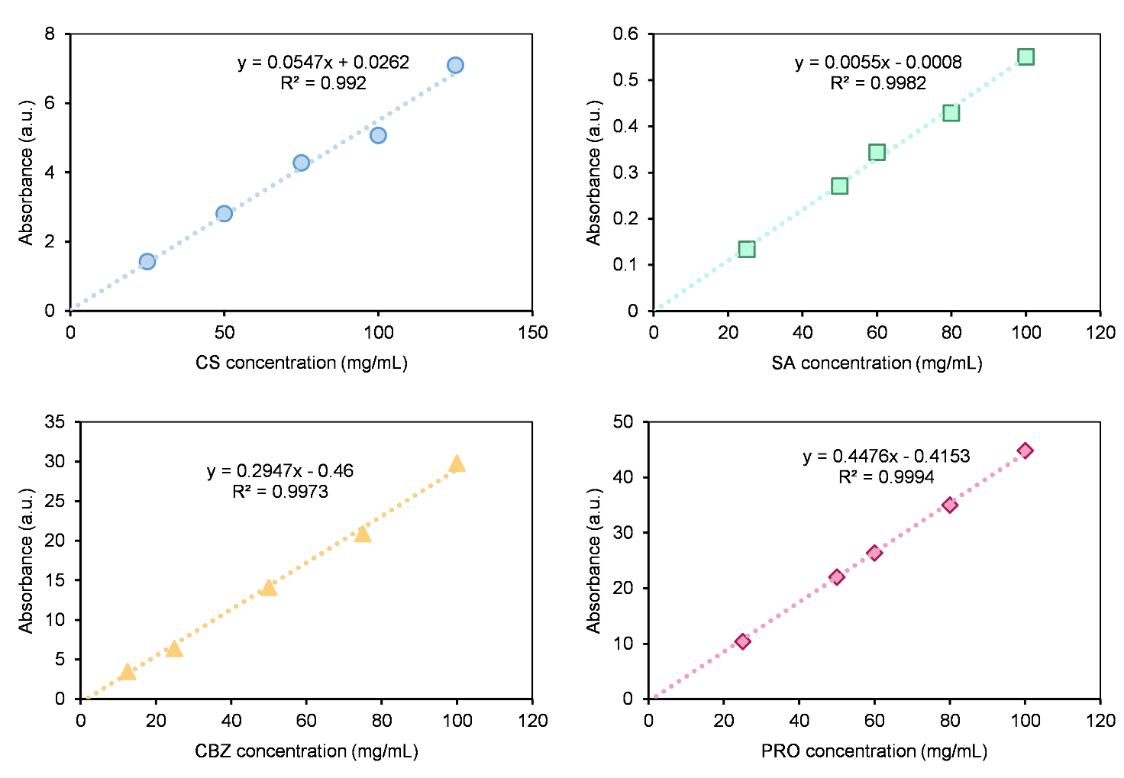
**

**Supplementary Figure 1 | Standard calibration curves for tested protectants.**


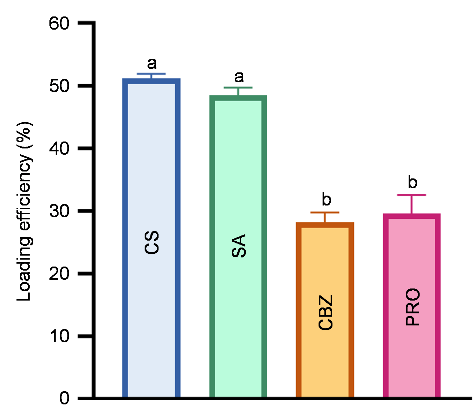


**Supplementary Figure 2 | Loading efficiencies of HLDP toward four types of protectants.** Different letters indicate significant differences according to the Brown-Forsythe test (*P* < 0.05). Each treatment contained three independent samples.


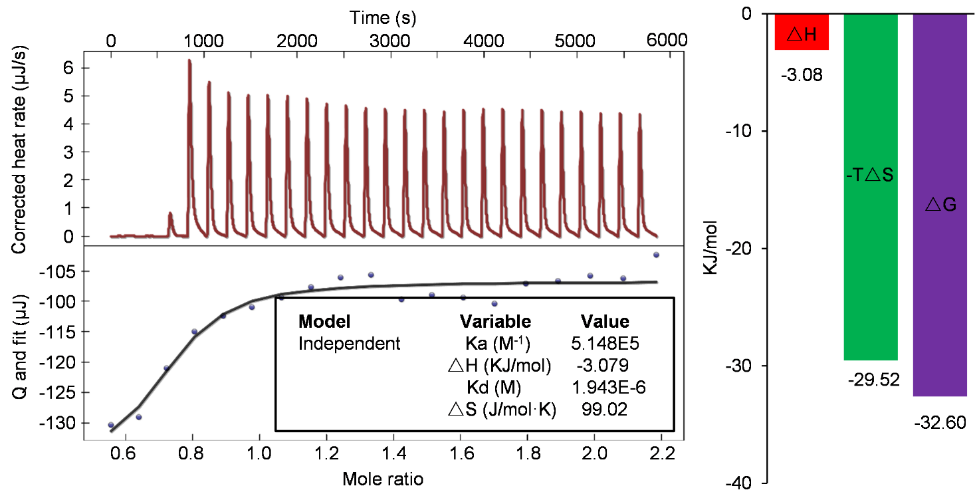


**Supplementary Figure 3 | Isothermal titration calorimetry of SA into HLDP.** The 1000 μL of HLDP solution (0.138 mM) was titrated with 250 μL of SA solution (1 mM). During each injection, the heating temperature of interaction was calculated by integrating each titration peak via Origin7 software, and ΔG value was calculated using the formula of ΔG = ΔH – TΔS.


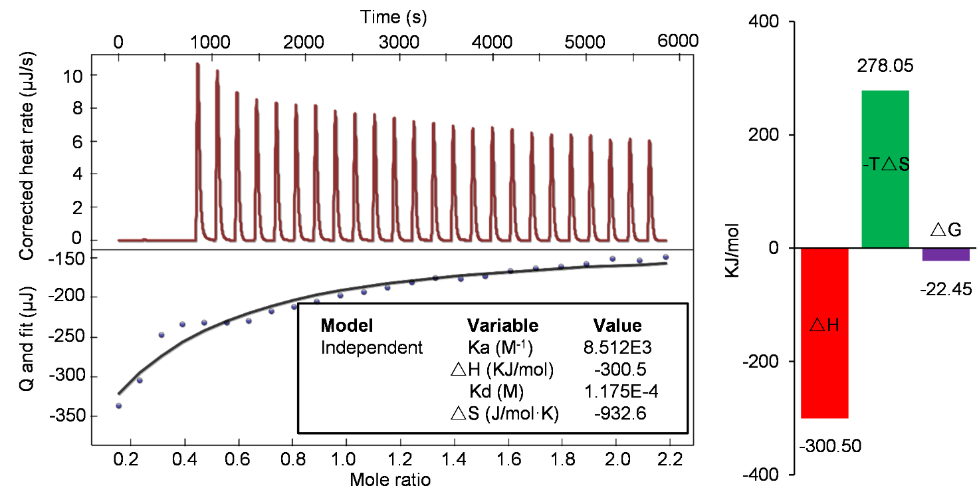


**Supplementary Figure 4 | Isothermal titration calorimetry of CBZ into HLDP.** The 1000 μL of HLDP solution (0.138 mM) was titrated with 250 μL of CBZ solution (1 mM). During each injection, the heating temperature of interaction was calculated by integrating each titration peak via Origin7 software, and ΔG value was calculated using the formula of ΔG = ΔH – TΔS.


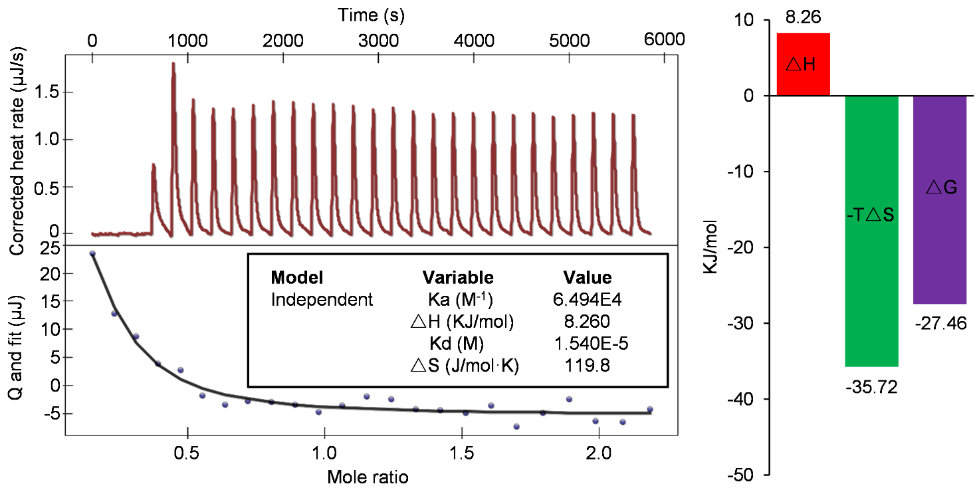


**Supplementary Figure 5 | Isothermal titration calorimetry of PRO into HLDP.** The 1000 μL of HLDP solution (0.138 mM) was titrated with 250 μL of PRO solution (1 mM). During each injection, the heating temperature of interaction was calculated by integrating each titration peak via Origin7 software, and ΔG value was calculated using the formula of ΔG = ΔH – TΔS.


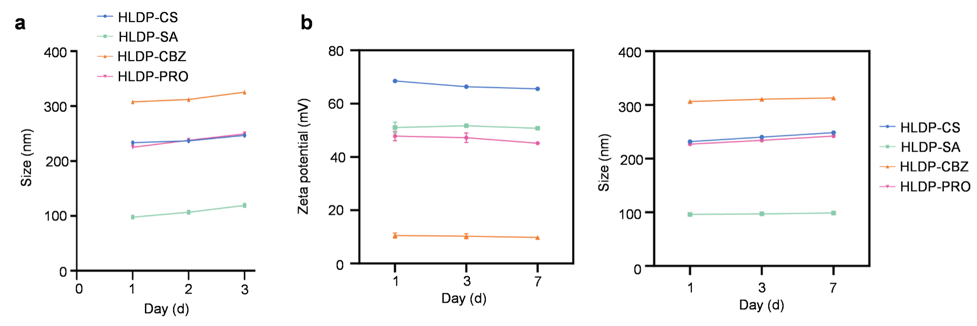


**Supplementary Figure 6 |** **Dissolution and stability of various HLDP nano-protectants. a,** Dissolution of various HLDP nano-protectants. HLDP nano-protectants were dissolved in ultrapure deionized water, and their particle sizes were measured at 1, 2 and 3 d under the oscillation condition. Each treatment included three independent samples. **b,** Stability of various HLDP nano-protectants. Zeta potentials and particle sizes of HLDP nano-protectants after the storage. Each treatment included three independent samples.


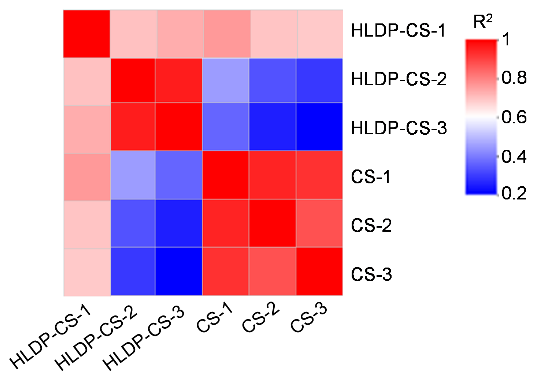


**Supplementary Figure 7 | Pearson correlation between collected samples.**


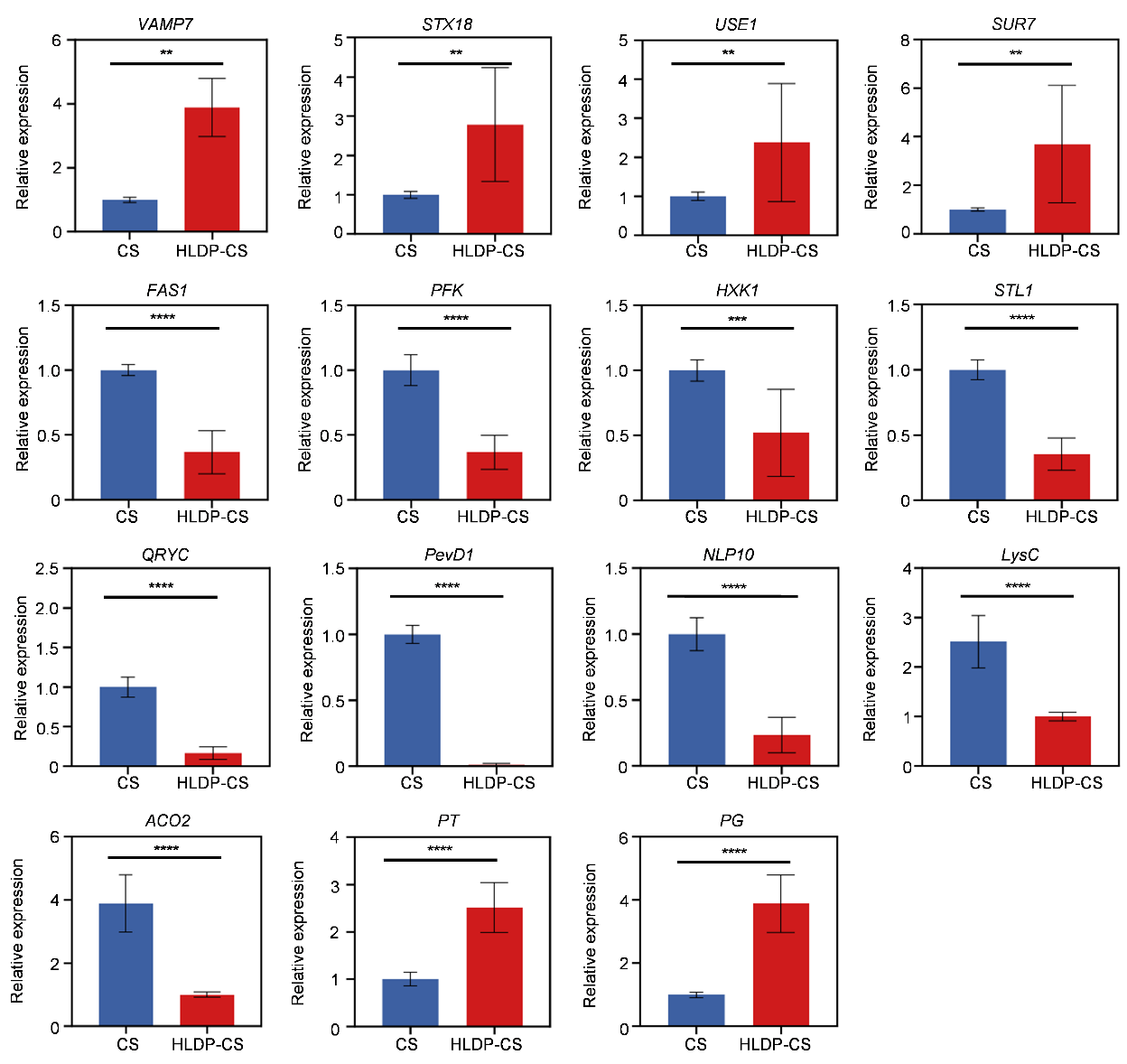


**Supplementary Figure 8 | Validation of differentially expressed genes using quantitative real-time polymerase chain reaction.** The relative expression level of each gene was normalized to the abundance of *GAPDH* gene. Each treatment included nine independent samples. The “**”, “***” and “****” indicate significant differences according to the ratio paired *t* test (*P* < 0.01, *P* < 0.001 and *P* < 0.0001), respectively.


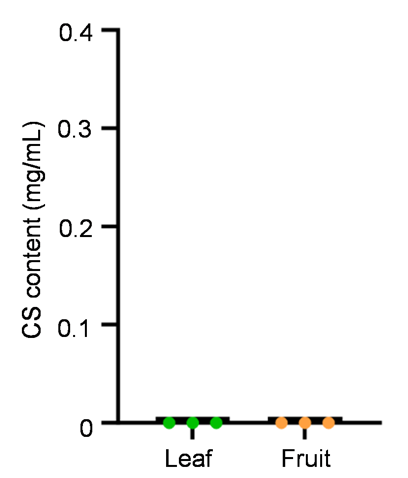


**Supplementary Figure 9 | CS contents in mango leaves and fruits treated with HLDP-CS nano-protectant.** Each treatment included three independent samples.


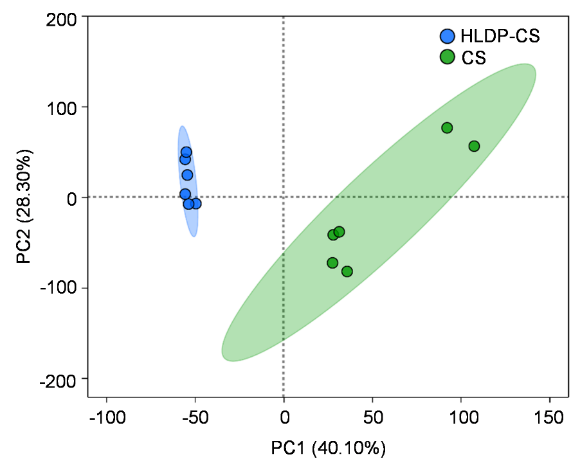


**Supplementary Figure 10 | Principal component analysis of collected samples.**


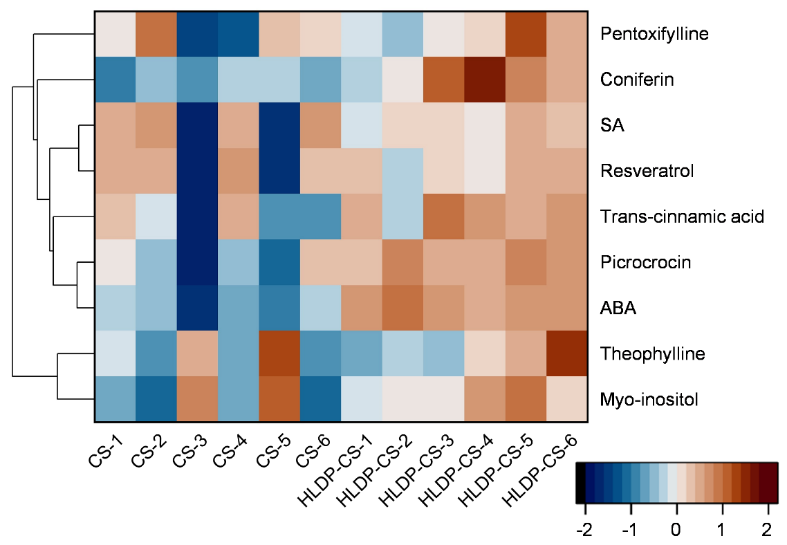


**Supplementary Figure 11 | Heatmap of differentially expressed metabolites in** **mango fruits treated with HLDP-CS nano-protectant and CS alone.** Highly and lowly expressed metabolites are labeled as red and blue, respectively. Each treatment included six independent samples.


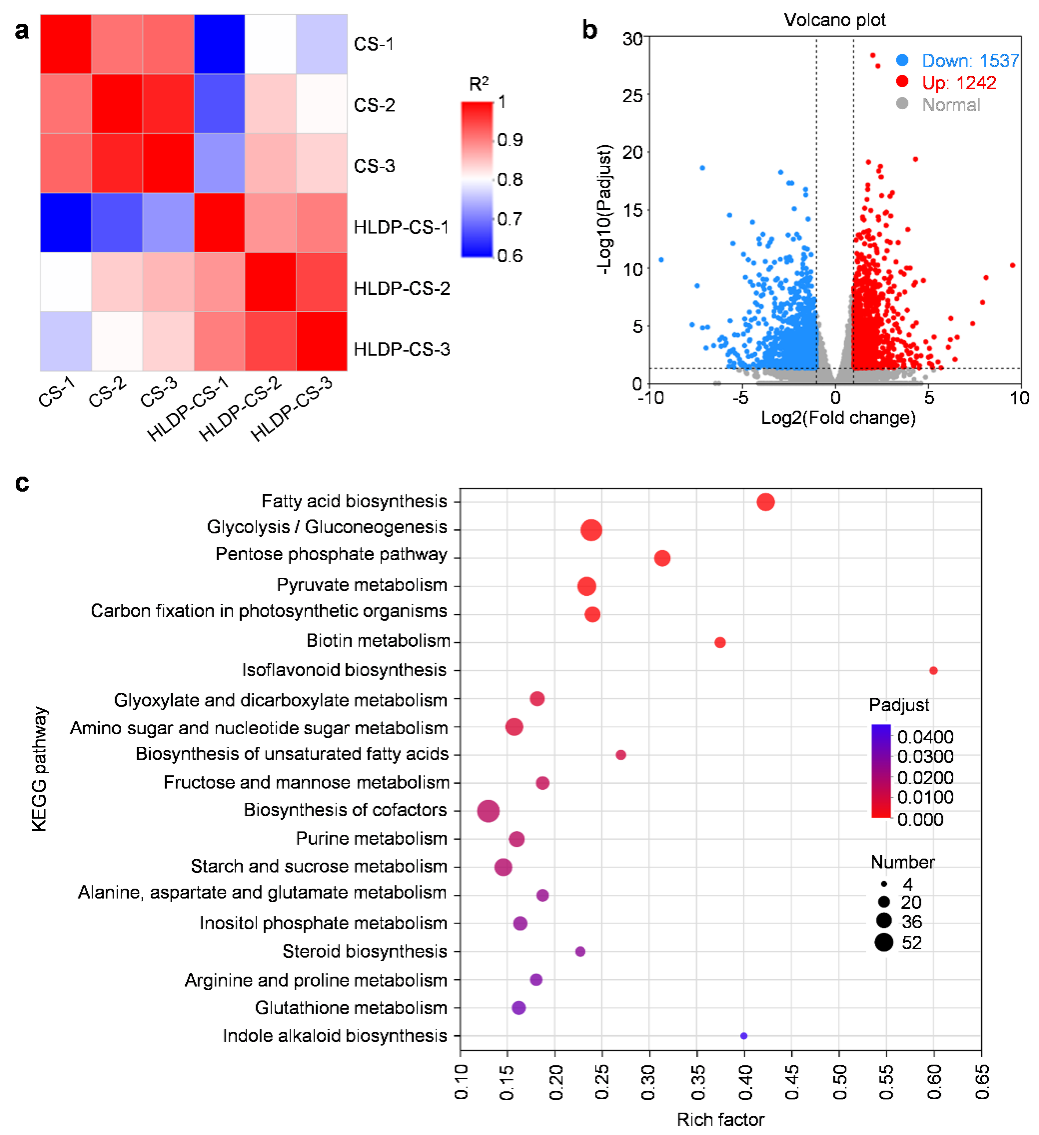


**Supplementary Figure 12 | RNA-seq analysis to illustrate the potential mechanism underlying amplified plant defence responses induced by HLDP-CS nano-protectant. a,** Pearson correlation between collected samples. **b,** Analysis of DEGs in mango fruits treated with HLDP-CS nano-protectant and CS alone with the volcano plot. Up- and down-regulated genes are represented by red and blue dots, respectively. **c,** KEGG enrichment of DEGs.


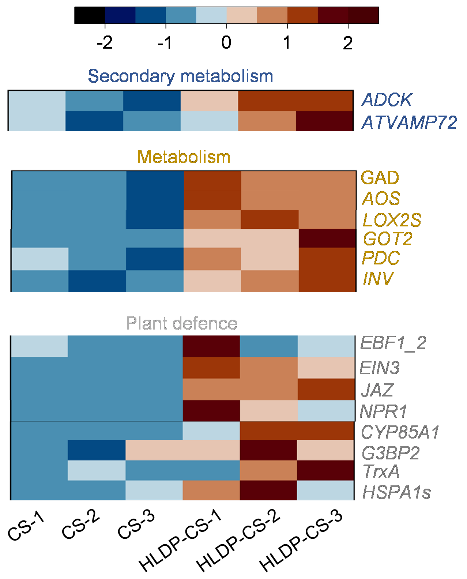


**Supplementary Figure 13 | Heatmap of various crucial genes related to plant defence responses.** Highly and lowly expressed genes are labeled as red and blue, respectively.


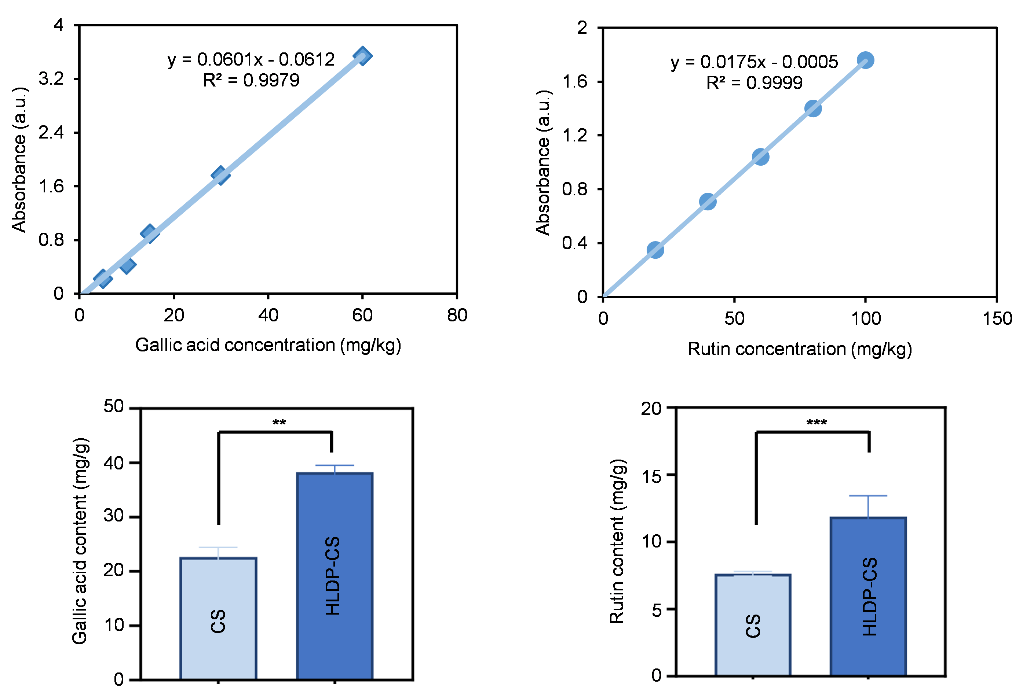


**Supplementary Figure 14 | Contents of gallic acid and rutin in mango fruits treated with HLDP-CS nano-protectant and CS alone.** Standard calibration curves for gallic acid and rutin are shown at the top. The contents of gallic acid and rutin are shown at the bottom. Each treatment included three independent samples. The “**” and “***” indicate significant differences according to the ratio paired *t* test (*P* < 0.01 and *P* < 0.001), respectively.


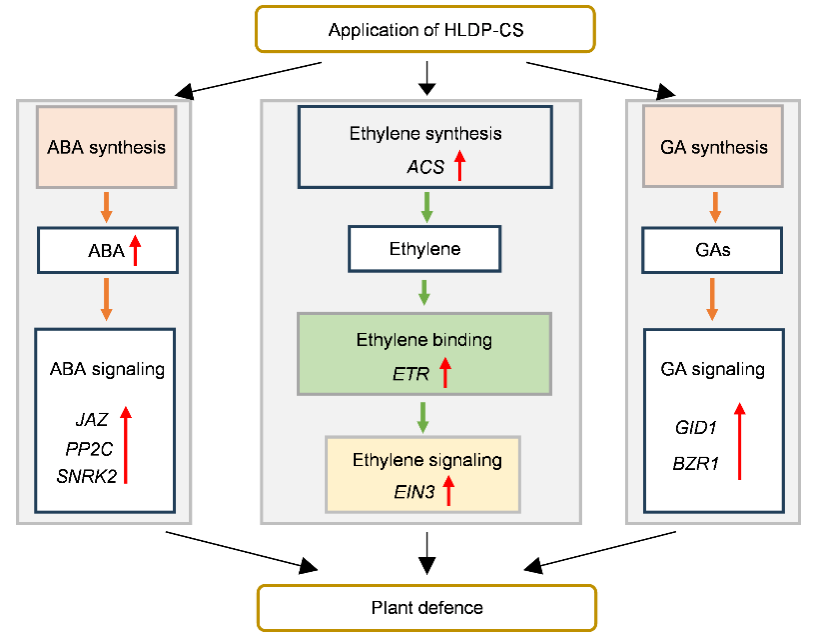


**Supplementary Figure 15 | Effects of HLDP-CS nano-protectant on plant hormone signaling pathway.** Up-regulated DEGs and DEMs are marked with red arrow. *JAZ*: Jasmonate-ZIM domain protein; *PP2C*: Protein phosphatase 2C; *SNRK2*: Sucrose nonfermenting-1-related kinase 2; *ACS*: 1-aminocyclopropane-1-carboxylic acid synthase; *ETR*: Ethylene receptor; *EIN3*: Ethylene-insensitive 3; *GID1*: Gibberellin insensitive dwarf 1; *BZR1*: Brassinazole-resistant 1.


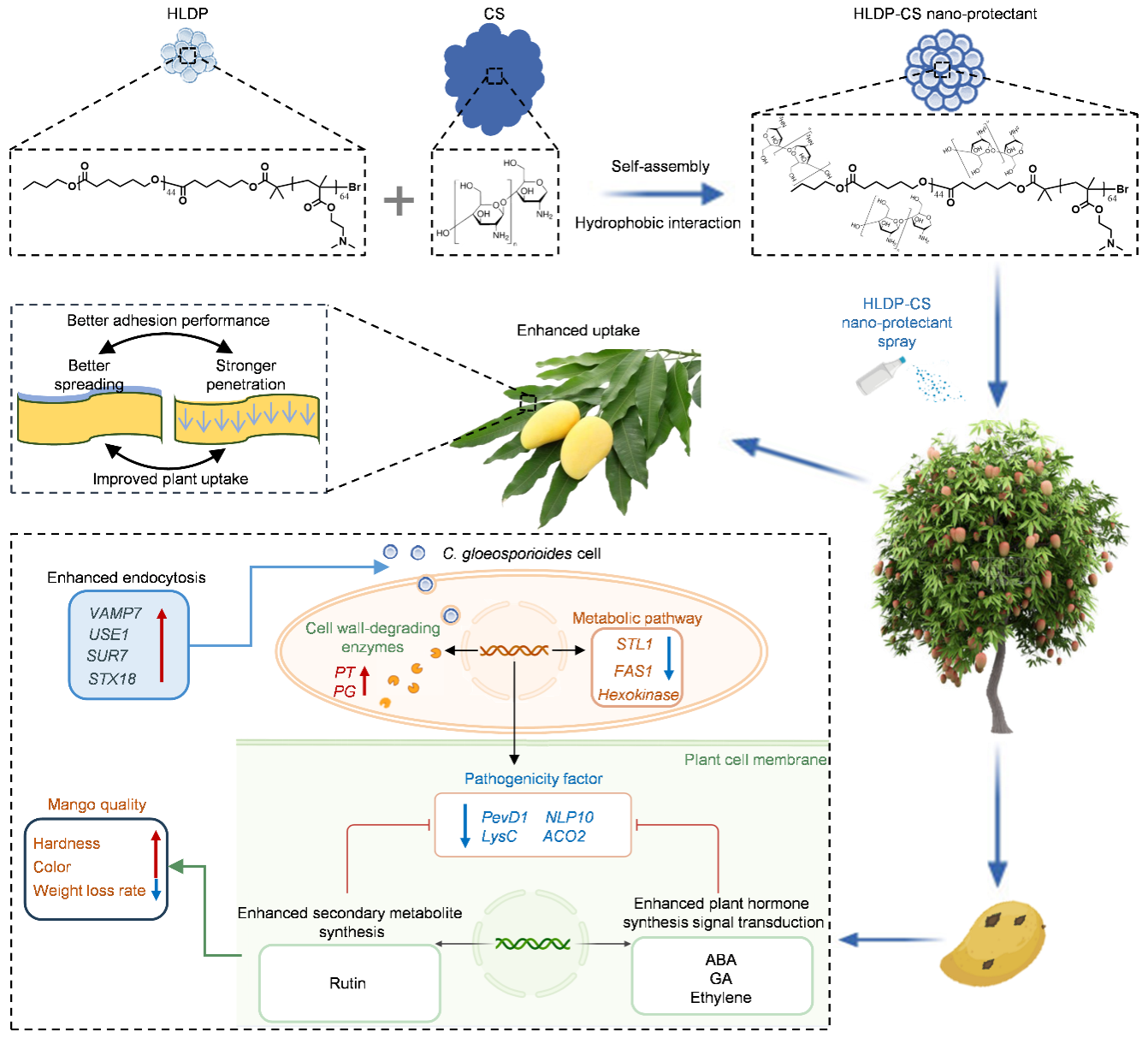


**Supplementary Figure 16 | Schematic illustration of HLDP-CS nano-protectant preparation and dual synergistic mechanism.** HLDP self-assembled with CS to form HLDP-CS nano-protectant via hydrophobic interaction. The efficiency green control of mango anthracnose was achieved via the direct pathogen inhibition and amplified plant defence responses.

**Supplementary Table 1 | Sequencing quality and genome mapping.**

| **Sample** | **Clean reads** | **Q20** | **GC percent** | **Percentage of mapped reads** |
| --- | --- | --- | --- | --- |
| CS-1 | 54484384 | 98.78 | 55.91 | 55.34% |
| CS-2 | 47437170 | 98.73 | 55.91 | 55.69% |
| CS-3 | 50543120 | 98.76 | 53.32 | 43.64% |
| HLDP-CS-1 | 40012642 | 98.80 | 55.03 | 56.47% |
| HLDP-CS-2 | 42709098 | 98.72 | 54.71 | 44.30% |
| HLDP-CS-3 | 47973516 | 98.75 | 54.17 | 44.55% |

**Supplementary Table 2 | Sequencing quality and genome mapping.**

| **Sample** | **Clean reads** | **Q20** | **GC percent** | **Percentage of mapped reads** |
| --- | --- | --- | --- | --- |
| CS-1 | 56352374 | 98.93 | 44.96 | 86.04% |
| CS-2 | 46600894 | 98.81 | 44.66 | 87.17% |
| CS-3 | 50394052 | 98.92 | 45.13 | 82.20% |
| HLDP-CS-1 | 47121666 | 98.91 | 45.65 | 73.92% |
| HLDP-CS-2 | 50058582 | 98.93 | 44.48 | 80.10% |
| HLDP-CS-3 | 41934796 | 98.67 | 49.43 | 58.04% |

**Supplementary Table 3 | Primers for qRT-PCR.**

| **Function** | **Gene** | **Primer** | **Sequence** |
| --- | --- | --- | --- |
| Fungal biomass | *LOC123201910* | Forward primer | ACTCCGTGTCACTGTCCAAGGT |
|  |  | Reverse primer | TGCTCTAGTGTGGCAGCCGTAA |
|  | *ACT3* | Forward primer | AGAGCTGTCTTCCGTAAGTCCC |
|  |  | Reverse primer | AGCACACGCCGGTTGATGACC |
| Transcriptome validation | *GAPDH* | Forward primer | AGGTCGGTGTGAACGGATTTG |
|  |  | Reverse primer | GGGGTCGTTGATGGCAACA |
|  | *VAMP7* | Forward primer | GCATCATGACCCGCAACATC |
|  |  | Reverse primer | CGCCATGACGATGAGGATGA |
|  | *STX18* | Forward primer | AAGTCAGGTACGTGGCATGG |
|  |  | Reverse primer | CTGCTCGATATGTGCCGACT |
|  | *USE1* | Forward primer | CTGGAGCAGGACAAGAACGT |
|  |  | Reverse primer | CAAAGACCAACAGCACCAGC |
|  | *SUR7* | Forward primer | ATTACGACGTGATCCGCCTC |
|  |  | Reverse primer | CAGTGGGAGATTCCGAGCTC |
|  | *FAS1* | Forward primer | CATCCGCAGCTACTACCAGG |
|  |  | Reverse primer | GCTCGTCGAAGTACTCCTCG |
|  | *PFK* | Forward primer | CCCTCCCTGTGCATTCCAAT |
|  |  | Reverse primer | ACTGGACCAGCTCGTTCAAG |
|  | *HXK1* | Forward primer | CCAATGGTCCAGCACTCCAT |
|  |  | Reverse primer | CGTAGCCTCGCAGGAGTAAG |
|  | *STL1* | Forward primer | TGCTGCCCTCTGTCTTGATG |
|  |  | Reverse primer | GATGATCTGGAGGAGCCACG |
|  | *ORYC* | Forward primer | CTATTGTACCGAGGTCGCCC |
|  |  | Reverse primer | CATCCAAACCACGATGGGGA |
|  | *PevD1* | Forward primer | CTCCTACGCCATCCGAGAAC |
|  |  | Reverse primer | TGAAGCGGTACTTGGACTCG |
|  | *NLP10* | Forward primer | GATCCCATAGGCACGACTGG |
|  |  | Reverse primer | CTCTCTGGGGGTTGTTGGAC |
|  | *LysC* | Forward primer | GCGAGCGAGATCAACGTTTC |
|  |  | Reverse primer | AGGACAGAGTCTTCGGCTCT |
|  | *ACO2* | Forward primer | ACGACGTCCATCTTCCCCTA |
|  |  | Reverse primer | GTTAATGTGGGGCTCCAGCT |
|  | *PT* | Forward primer | AAGACCTCCCCGACGGATAA |
|  |  | Reverse primer | CAGAGTCAACCCACCACTCC |
|  | *PG* | Forward primer | TGGAACAAAGACTCCGTCGG |
|  |  | Reverse primer | CGTCGCAGCCTTTGATGATG |
